# Supplementary material for: Monolithic FAPbBr3 photoanode for photoelectrochemical water oxidation with low onset-potential and enhanced stability
Source: Nat Commun. 2023 Sep 7;14:5486. doi: 10.1038/s41467-023-41187-9 (PMC10484934; doi:10.1038/s41467-023-41187-9)
Supplement: Supplementary file 1 — Supplementary Information [file 41467_2023_41187_MOESM1_ESM.pdf]

Supplementary Information

Monolithic $FAPbBr_3$ Photoanode for Photoelectrochemical Water Oxidation with Low Onset-potential and Enhanced Stability

Hao Yang,[1†] Yawen Liu,[2†] Yunxuan Ding,[3] Fusheng Li,[4] Linqin Wang,[3] Bin Cai,[2] Fuguo Zhang,[1] Tianqi Liu,[1] Gerrit Boschloo,[2] Erik M. J. Johansson,[2*] and Licheng Sun[1,3,4*]

1) Department of Chemistry, School of Engineering Sciences in Chemistry, Biotechnology and Health, KTH Royal Institute of Technology, 10044 Stockholm, Sweden
2) Department of Chemistry-Ångström, Physical Chemistry, Uppsala University, 75120 Uppsala, Sweden
3) Center of Artificial Photosynthesis for Solar Fuels and Department of Chemistry, School of Science, Westlake University, 310024 Hangzhou, China
4) State Key Laboratory of Fine Chemicals, Institute of Artificial Photosynthesis, DUT-KTH Joint Education and Research Centre on Molecular Devices, Dalian University of Technology, 116024 Dalian, China

**Corresponding authors: lichengs@kth.se; erik.johansson@kemi.uu.se*
†These authors contributed equally to this work.

Supplementary Methods

Physical characterizations

The current density–voltage curves (*J*-*V*) of the as-fabricated solar cell were measured by Wave Labs SINUS-70 solar simulator with ultraviolet (UV) and infrared (IR) range extenders (100 mW cm^{-2}). The aperture area of the black metal mask used in the *J*-*V* measurements was 0.125 cm^2 unless further mentioned. The incident photon-to-current efficiency (IPCE) spectra of solar cells were measured by a homemade two-electrode setup consisting of a monochromator (Spectral products CM110), a Xenon lamp (Spectral Products ASB-XE-175), and a LabJack U6 data acquisition board. The IPCE spectra of photoanodes were measured using a homemade three-electrode setup consisting of a monochromator (Jobin Yvon HORIBA H10-61 UV), a Xenon lamp (NEWPORT LCS-100 solar simulator 94011A-ES), a Si photodiode photometer (Thorlabs, S130VC) and a CHI 660E electrochemical workstation. Steady-state photoluminescence (PL) was obtained by a PL spectrometer (Edinburgh Instruments, FLS 980). The UV-vis spectra were recorded by an HR-2000 Ocean Optics spectrophotometer with baseline correction. Water contact angle analysis was carried out using a contact angle goniometer from Ossila. X-ray diffraction (XRD) measurements were performed on a Siemens D5000 θ−2θ goniometer with Cu Kα (λ = 1.54051 Å) radiation and a 0.4° Soller slit collimator, which had a resolution of 2θ = 0.3° (Bruker AXS, Karlsruhe, Germany) at room temperature. The morphology and composition of the fabricated films were characterized using ZeissLEO1530/1550 scanning electron microscope (SEM) and an energy-dispersive X-ray spectroscopy (EDS) detector (Oxford AZtec EDS system). The surface chemical state of electrode films was investigated using X-ray photoelectron spectroscopy (XPS) on a PHI Quantera II from Physical Electronics. The ultraviolet photoelectron spectroscopy (UPS) spectra were acquired using an Thermo fisher ESCALAB Xi^+ with excitation provided by the He I emission line (21.2 eV). The infrared spectra of the fabricated films were characterized by a Bruker Vertex 70v Fourier transform infrared (FT-IR) spectrometer by the sampling methodology of Attenuated total reflectance (ATR) with background correction. Faraday efficiency measurements were carried out with an Omega PXM409 pressure transducer and a laboratory-made H-cell. Infrared images were taken by thermal imager with thermal sensitivity of 40 mK (Testo 883).

Supplementary Figures, Tables and Notes

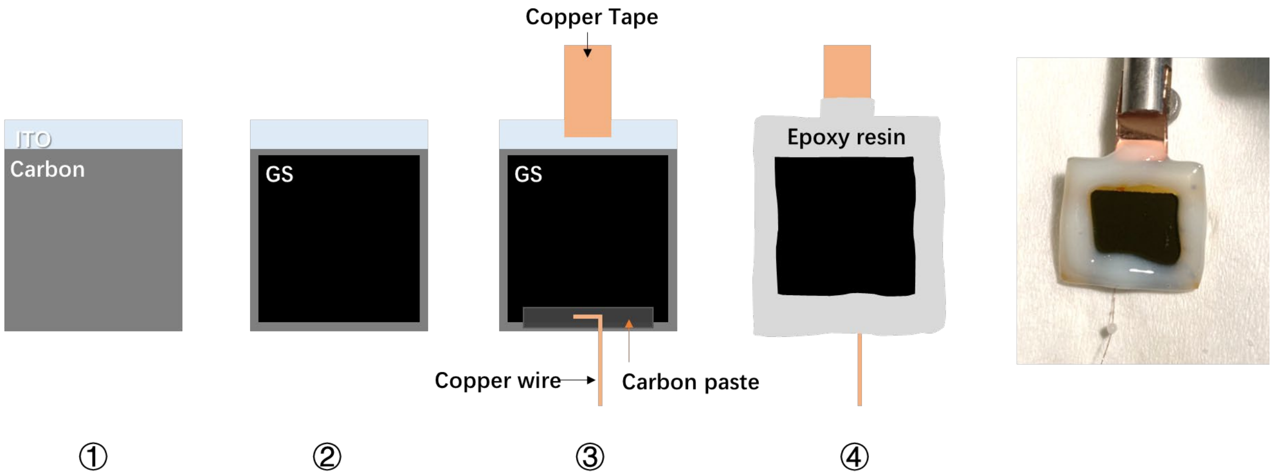

Supplementary Fig. 1 Schematic diagram of photoanode preparation in top view.

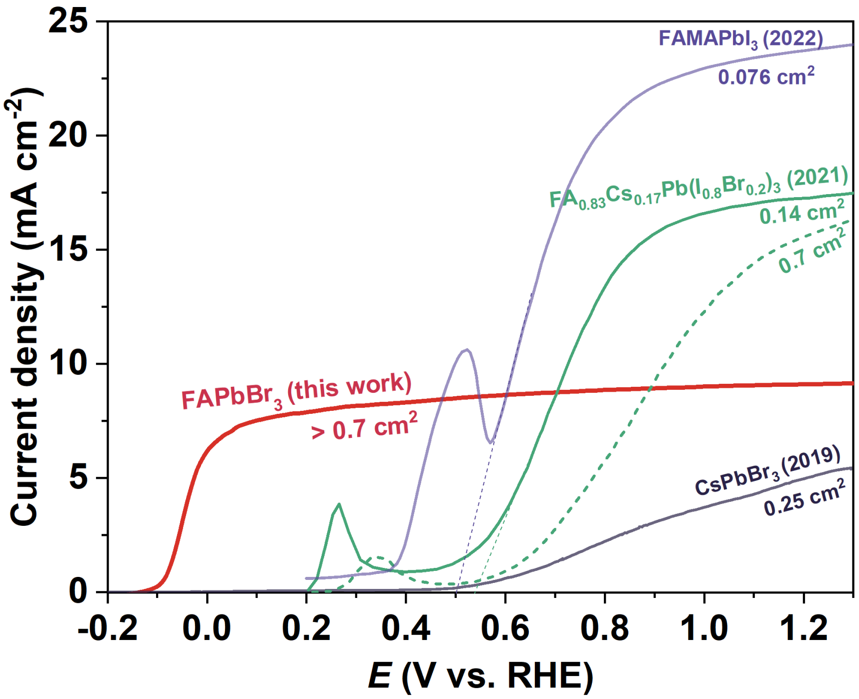

Supplementary Fig. 2 Comparison of the linear sweep voltammetry (LSV) curves between the $FAPbBr_3$ photoanode and other perovskite photoanodes made from a single junction absorber layer including $CsPbBr_3$,[1] $FAMAPbI_3$,[2] and $FA_{0.83}Cs_{0.17}Pb(I_{0.8}Br_{0.2})_3$.[3] The LSV data was extracted from corresponding literature.

Supplementary Note 1: Preparation and characterization of light-absorbing layers.

The high bandgap $FAPbBr_3$ perovskite photoanode is composed of a light-absorbing part and a water oxidation catalyst (WOC) part. The device structure of the light-absorbing part (i.e., $FAPbBr_3$ solar cell) is schematically shown in ***Supplementary Fig. 3a***. In the as-fabricated hole transport material (HTM)-free $FAPbBr_3$ solar cell, $FAPbBr_3$ layer is sandwiched between a compact SnO_2 layer and a conductive mesoporous carbon layer. ***Supplementary Fig. 4*** presents the elemental mapping images of the $FAPbBr_3$ solar cell, which clearly show the uniform distribution of related elements in each functional layer. ***Supplementary Fig. 3b*** presents the scanning electron microscopy (SEM) cross-section image of the HTM-free $FAPbBr_3$ solar cell; the perovskite layer thickness is around 400 nm. As shown in ***Supplementary Fig. 3c***, the top view SEM image shows high-quality perovskite film without pinholes; the $FAPbBr_3$ large crystals are compactly stacked, indicating high crystallinity quality and few boundary defects. The surface of the perovskite film was examined by multiple techniques, including using UV−vis absorption spectra, X-ray Photoelectron spectroscopy (XPS), X-ray diffraction (XRD), and steady-state photoluminescence (SSPL), all the results correspond with our previous reports[4, 5] and demonstrate the high quality of as-prepared perovskite film. The UV–vis absorption spectrum of the $FAPbBr_3$ film shows sharp absorption peaks at the band edge near 530 nm in ***Supplementary Fig. 5a***; the absorption edge is over 550 nm, indicating an optical band gap of around 2.25 eV. The crystal structure of as-fabricated $FAPbBr_3$ films was measured using XRD. As shown in ***Supplementary Fig. 5b***, the absence of $PbBr_2$ peak indicate the complete conversion of $PbBr_2$ to perovskite phase; the sharp main diffraction peaks of the $FAPbBr_3$ (located at 14.8° and 29.7°) suggest good crystallinity of the perovskite films. The SSPL spectrum shows a strong peak centered at 545 nm, which implies a lower density of defect states in the perovskite film (***Fig. S4c***). The XPS survey spectrum is present in ***Supplementary Fig. 3d***; all the element from $FAPbBr_3$, including C, N, Pb, and Br, is clearly identified.

From the *J-V* curves in ***Supplementary Figs. 3d***, ***6a,*** and ***Supplementary Table 1***, the champion device shows a power conversion efficiency (PCE) of 9.16% with a high open-circuit voltage (V_{oc}) of 1.38 V, a short-circuit current density (J_{sc}) of 8.69 mA cm^{-2}, and fill factor (FF) of 76.04%. The performance is even superior to the reference device using Spiro-MeOTAD as

HTM (***Supplementary Table 2***), which demonstrates the promising potential for the construction of low onset potential photoanode based on simple HTM-free configuration. The photovoltaic performance distributions for multiple devices are shown in ***Supplementary Fig. 7***, demonstrating a higher reproducibility of the carbon samples than gold samples. The device shows high IPCE over 70% from wavelengths 350 to 550 nm, leading to an integral current density near 7.4 mA cm^{-2}, which is 14% less than the value of J_{sc} extracted from the *J-V* curve (***Supplementary Fig. 3e***). The discrepancy between the measured J_{sc} and integrated J_{sc} was also reported in other $FAPbBr_3$ solar cells[6], and may be due to different illumination intensity for the IPCE measurement compared to the *J-V* measurement. Typically, perovskite solar cells with similar carbon-based structures show good stability in humid conditions due to the hydrophobic nature of the mesoporous carbon layer.[7] ***Supplementary Fig. 3f*** shows the water contact angle of the $FAPbBr_3$/Carbon, $FAPbBr_3$/Au and pristine $FAPbBr_3$ film; $FAPbBr_3$/Carbon exhibits water contact angle (WCA) of 104°, which is apparently large than gold sample (61.9°) and pristine $FAPbBr_3$ film (43.2°). It is worth mentioning that the $FAPbBr_3$ film decomposed immediately after contact with water, leading to a decreased WCA with time. With the protection of the hydrophobic carbon layer, the $FAPbBr_3$/Carbon device showed 106% of its original efficiency under dry-air conditions after 300 days (***Supplementary Fig. 3g***). ***Supplementary Fig. 8*** and ***Supplementary Table 3*** show the performance of fresh and aged samples: V_{oc} and FF parameters remain unchanged after 150 days; J_{sc} is even improved, which leads to the enhancement of PCE after 300 days of treatment. The photo-stability of $FAPbBr_3$/Carbon was evaluated under continuous one-sun illumination (open circuit conditions): no performance decay was observed after 72 hours of illumination; the device kept 73% of its original efficiency even after 160 hours of illumination (***Supplementary Fig. 3h***). The corresponding ambient thermal stability was investigated under 85°C heating conditions. As shown in ***Supplementary Fig. 3i***, the device PCE degraded to 80% after 72 hours of operation, which further decreased to 65% efficiency after 160 hours of heating. The high voltage, excellent stability, and hydrophobic nature of HTM-free $FAPbBr_3$/Carbon devices are beneficial to the building of $FAPbBr_3$-based photoelectrochemical water oxidation devices.

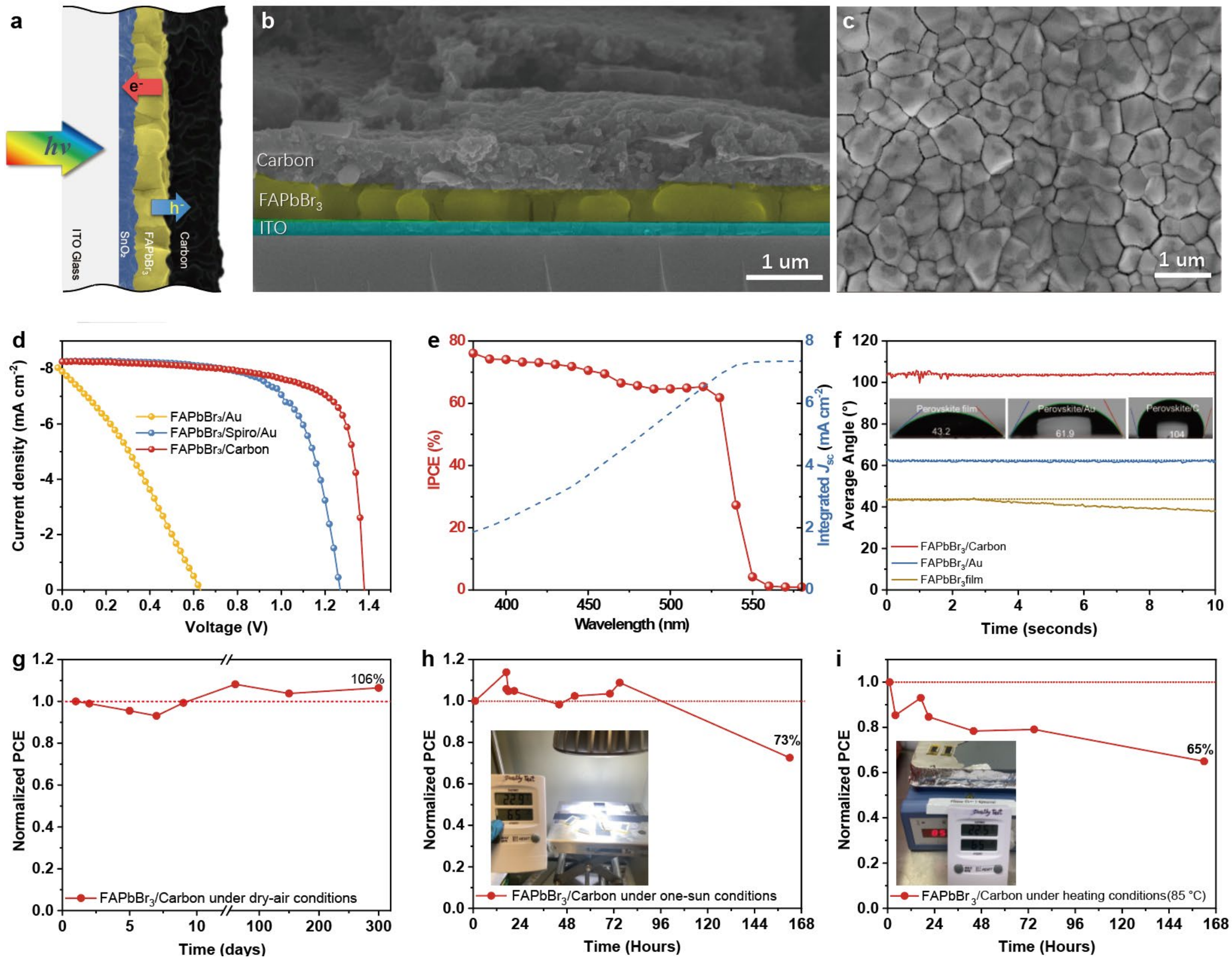

Supplementary Fig. 3 Assessing and comparing $FAPbBr_3$ solar cell performance. (a) Device structure of HTM-free $FAPbBr_3$ solar cell. SEM images of HTM-free $FAPbBr_3$/Carbon solar cell in (b) cross-section view and (c) top view. (d) Representative *J*-*V* curves of $FAPbBr_3$/Carbon solar cell and reference solar cells. (e) IPCE spectrum of $FAPbBr_3$/Carbon solar cell. (f) Water contact angle analysis of $FAPbBr_3$/Carbon solar cell film and other reference films. Stability tests of $FAPbBr_3$/Carbon solar cell under (g) dry-air conditions, (h) one-sun conditions, and (i) 85°C heating conditions.

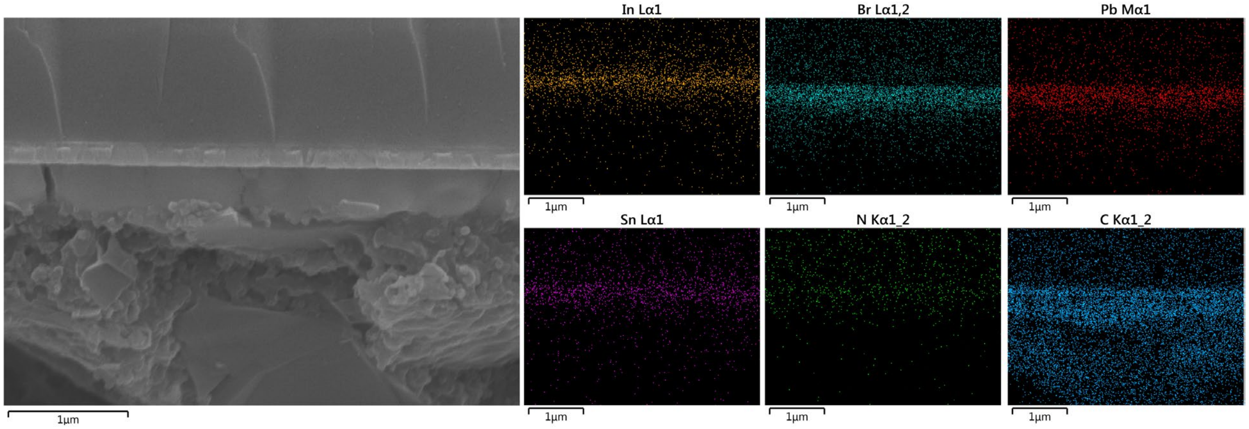

Supplementary Fig. 4 Side-view SEM and elemental mapping images of $FAPbBr_3$ solar cell.

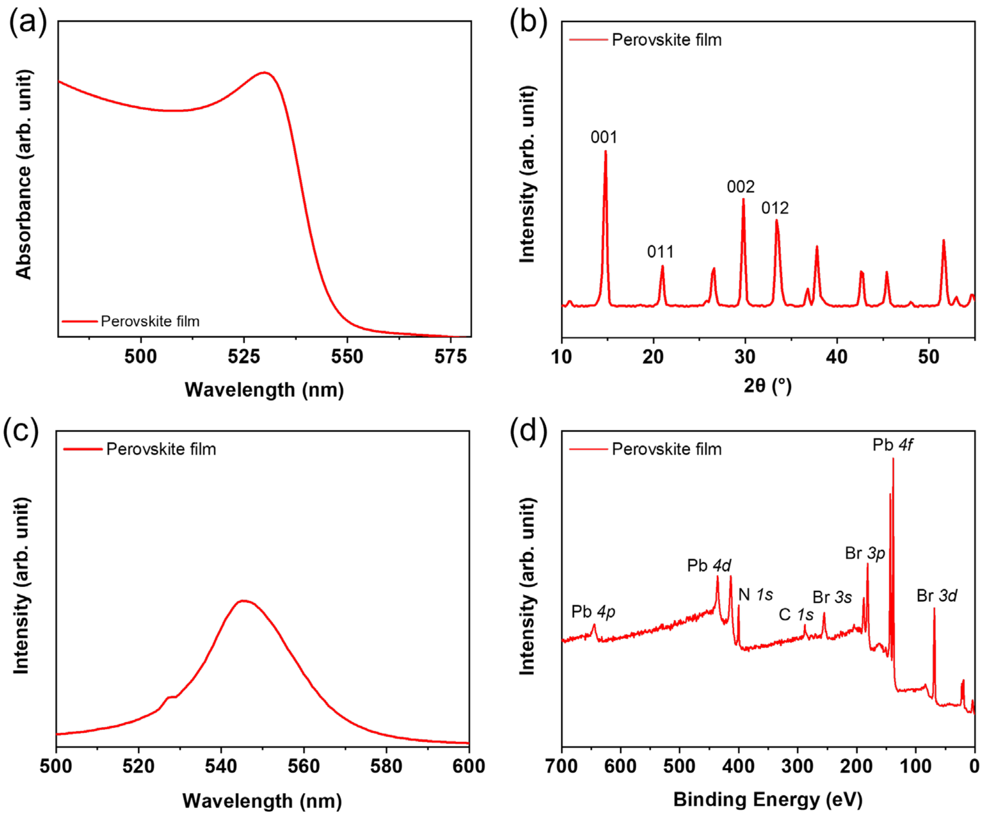

Supplementary Fig. 5 Spectroscopic characterization of $FAPbBr_3$ perovskite films. (a) UV–vis spectrum. (b) XRD pattern. (c) SSPL. (d) XPS survey spectrum.

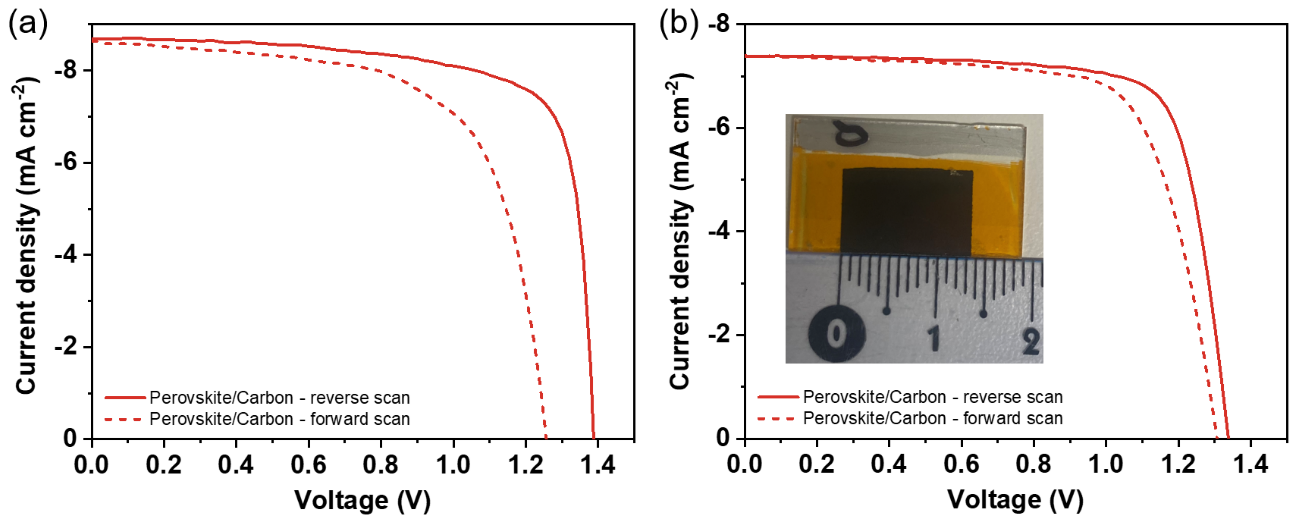

Supplementary Fig. 6 Representative *J-V* curves of $FAPbBr_3$/Carbon solar cell with (a) small exposure area (0.125 cm^{-2}) and (b) large exposure area (1.0 cm^{-2}).

Supplementary Table 1 Device parameters for representative $FAPbBr_3$/Carbon cell.

Device	PCE (%)	FF (%)	J_{sc} ($mA.cm^{-2}$)	V_{oc} (V)
$FAPbBr_3$/Carbon Reverse	9.16	76.04	−8.69	1.38
$FAPbBr_3$/Carbon Forward	7.07	65.33	−8.61	1.26
Large size-$FAPbBr_3$/Carbon Reverse	7.59	76.55	−7.40	1.34
Large size-$FAPbBr_3$/Carbon Forward	6.90	71.16	−7.40	1.31

Supplementary Table 2 Device parameters for reference cell.

Device	PCE (%)	FF (%)	J_{sc} ($mA.cm^{-2}$)	V_{oc} (V)
$FAPbBr_3$/Au	1.53	30.79	−7.87	0.63
$FAPbBr_3$/Spiro/Au	7.13	67.97	−8.26	1.27

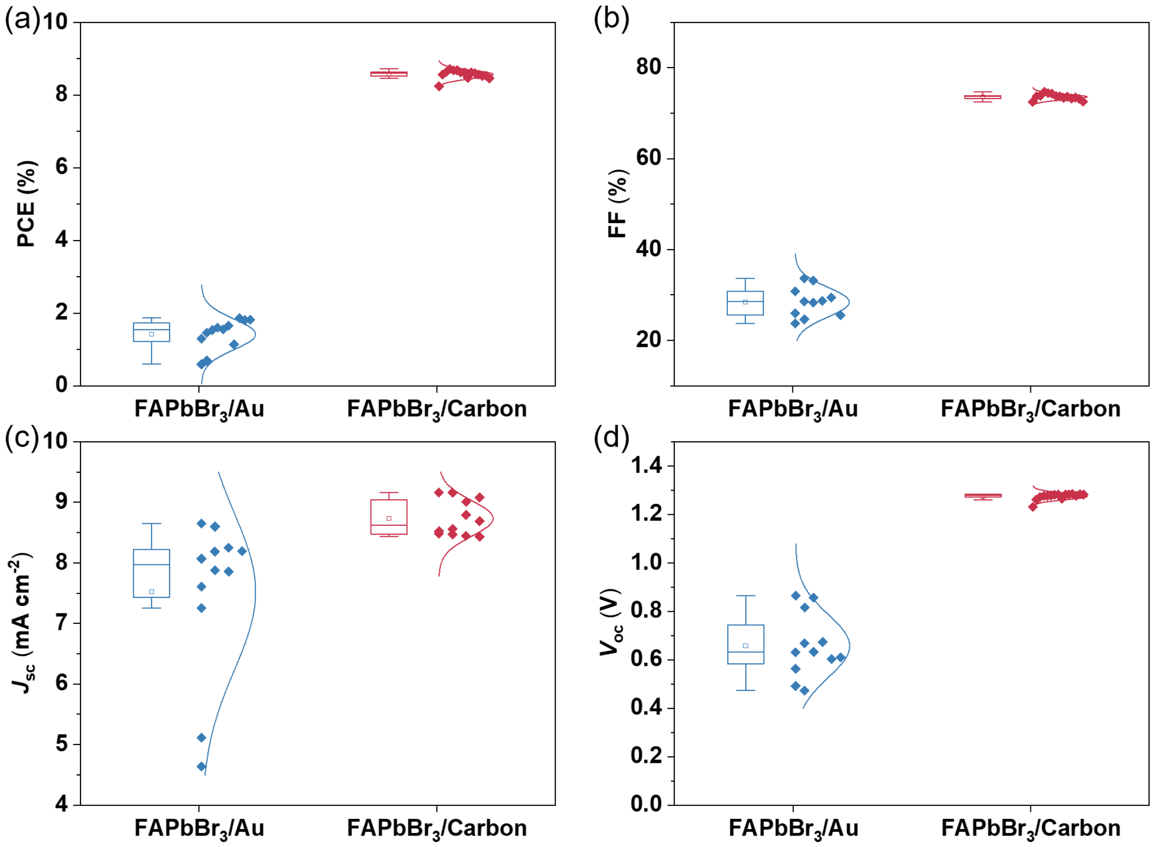

Supplementary Fig. 7 Statistic photovoltaic parameters of (a) PCE, (b) FF, (c) J_{sc}, and (d) V_{oc} of $FAPbBr_3$/Carbon solar cell and $FAPbBr_3$/Au solar cell.

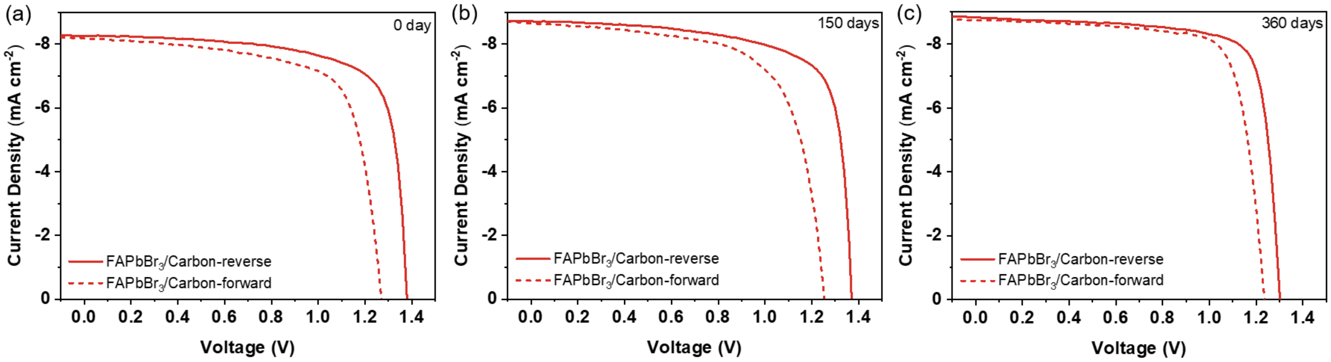

Supplementary Fig. 8 Representative *J-V* curves of $FAPbBr_3$/Carbon solar cell. (a) pristine, aged for (b) 150 days and (c) 300 days under dry-air conditions.

Supplementary Table 3 Device parameters for pristine and aged (dry-air conditions) $FAPbBr_3$/Carbon solar cells.

Device	PCE (%)	FF (%)	J_{sc} (mA.cm^{-2})	V_{oc} (V)
Pristine $FAPbBr_3$/Carbon Reverse	8.47	74.44	−8.26	1.38
Pristine $FAPbBr_3$/Carbon Forward	7.29	70.23	−8.18	1.27
Aged (150 days) $FAPbBr_3$/Carbon Reverse	8.79	73.60	−8.74	1.37
Aged (150 days) $FAPbBr_3$/Carbon Forward	7.20	66.33	−8.65	1.26
Aged (300 days) $FAPbBr_3$/Carbon Reverse	9.02	78.42	−8.83	1.30
Aged (300 days) $FAPbBr_3$/Carbon Forward	8.26	76.38	−8.75	1.24

Supplementary Note 2: Preparation and characterization of water oxidation catalyst layers.

Nickel iron (NiFe) composition has been widely reported as an efficient electrocatalyst for water oxidation. In our system, a compact attached NiFe alloy layer was first deposited on GS substrate using an electrodeposition method[8] to improve the adhesion of oxides catalyst. Then, active NiFe layer double hydroxide (LDH) was electro-deposited[9] onto the GS/NiFe alloy to create a stable and highly active GS/NiFe alloy/NiFe LDH part (***Supplementary Fig. 9a***). ***Supplementary Fig. 9b*** shows that the GS/NiFe alloy/NiFe LDH catalyst exhibits the highest catalytic current compared to the GS/NiFe alloy and GS/NiFe LDH electrodes. Moreover, the overpotential requirement was significantly reduced in the composite GS/NiFe alloy/NiFe LDH electrode, with only 228 mV required to reach a current density of 10 mA cm^{-2}, which is 61 and 69 mV lower than that of the GS/NiFe LDH and GS/NiFe alloy electrodes, respectively. ***Supplementary Fig. 9c*** presents the Tafel slopes of the three aforementioned electrodes, which are around 30 to 40 mV dec^{-1}, suggesting the presence of similar active NiFe species on the surface of electrodes. The inset figure in ***Supplementary Fig. 9b*** displays the linear sweep voltammetry (LSV) curves in the $Ni^{2+/3+}$ redox region, revealing that the oxidation peak of the GS/NiFe alloy/NiFe LDH electrode is significantly larger than that of the GS/NiFe LDH and GS/NiFe alloy electrodes. Thus, the enhanced current observed in the GS/NiFe alloy/NiFe LDH electrode is attributed to the higher loading amount of active species. The catalytic stability of the electrodes was evaluated by chronopotentiometry under a constant current density of 10 mA cm^{-2}. As shown in ***Supplementary Fig. 9e***, GS/NiFe alloy/ NiFe LDH electrode exhibits the best stability with only a 19 mV increase in overpotential after 100 hours of catalysis. In contrast, the overpotential increase of the GS/NiFe LDH electrode was 81 mV. Additionally, black precipitates were observed at the bottom of the electrolyzer, which indicated that the loosely stacked hydroxide was prone to peel off from the glossy graphite surface (***Supplementary Fig. 10***). The amount of oxygen electrochemically generated from the GS/NiFe alloy/ NiFe LDH was confirmed using a pressure transducer. Quantitative yields of 97.0% and 99.3% are obtained under 5 and 10 mA cm^{-2} in 1.0 M KOH, respectively, which indicates that accumulated charge is nearly quantitatively consumed in oxygen evolution reaction (OER) (***Supplementary Figs. 9d*** and ***11***).

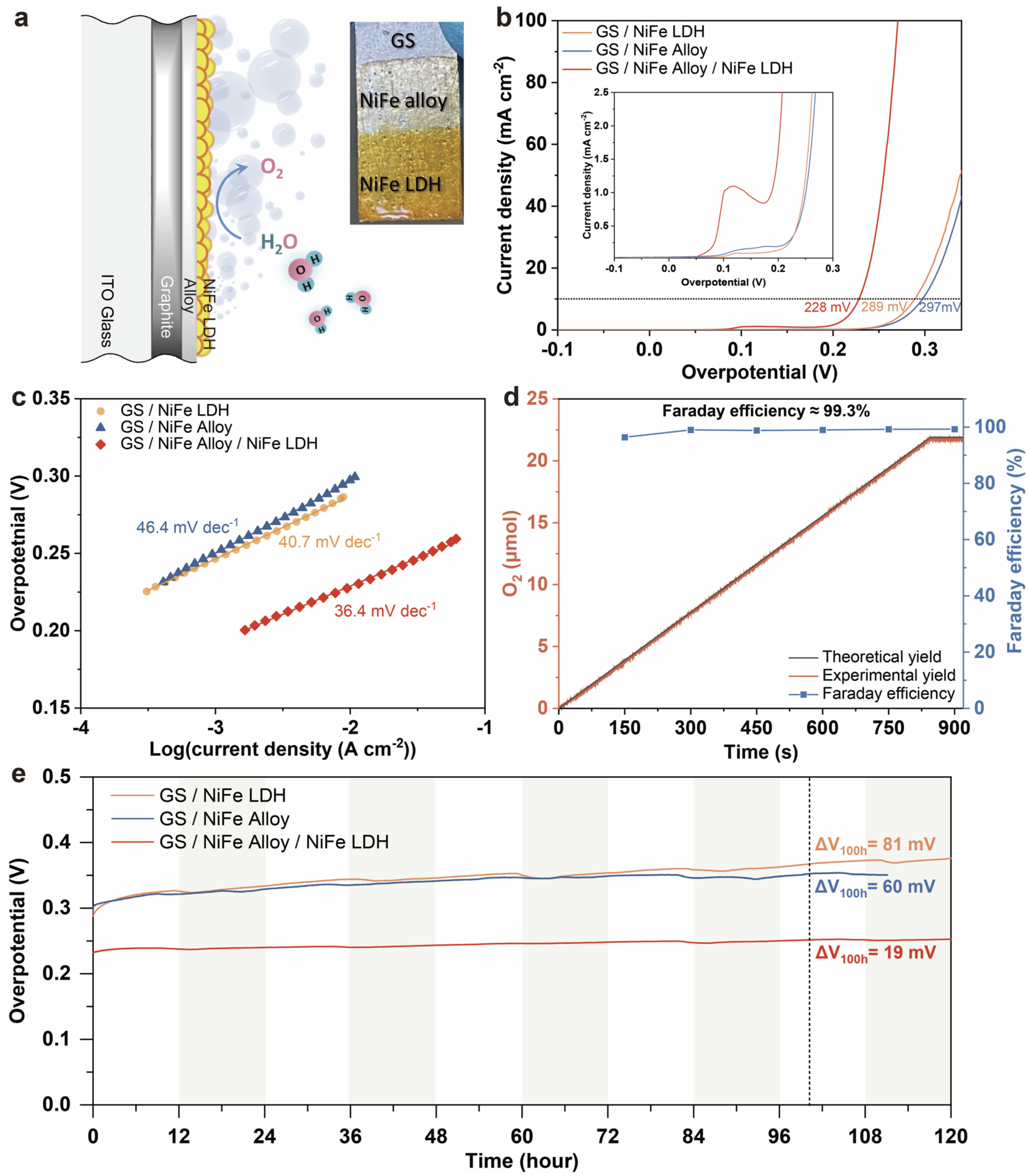

Supplementary Fig. 9 Catalytic performance of water oxidation catalyst layers. (a) Device structure of water oxidation catalyst layer. Inset: digital image of as-prepared catalyst layers. (b) LSV curves of GS/NiFe alloy/NiFe LDH, GS/NiFe alloy, and GS/NiFe LDH in 1.0 M KOH (scan rate: 1 mV s^{-1}). Inset: LSV curves in $Ni^{2+/3+}$ redox region. (c) Corresponding Tafel slopes and (d) Faradaic efficiencies of GS/NiFe alloy/NiFe LDH for the OER in 1.0 M KOH. Theoretically calculated and experimentally measured amounts of O_2 were shown as functions of electric charge for water oxidation catalyzed by

GS/NiFe alloy/NiFe LDH at a current density of 10 mA cm^{-2} (electrode area = 1.0 cm^2). (e) Chronopotentiometry measurement (10 mA cm^{-2}) of GS/NiFe alloy/NiFe LDH, GS/NiFe alloy and GS/NiFe LDH in 1.0 M KOH.

The surface morphology was characterized by SEM. As shown in ***Supplementary Fig. 12***, NiFe alloy could be uniformly deposited onto the graphite layer with no visible cracks. However, electroplated NiFe LDH could not format dense layers when deposited directly onto graphite; the isolated NiFe LDH "islands" reduced the attachment strength and loading amount, leading to poor mechanical strength and catalytic current. After the NiFe alloy layer was coated onto the graphite, a dense NiFe LDH layer could be further obtained by electrodeposition, resulting in the GS/NiFe alloy/ NiFe LDH electrode being the most efficient and stable. The SEM images of the electrodes after 120 hours of electrolysis are presented in ***Supplementary Fig. 13***. For GS/NiFe LDH electrode, most NiFe LDH flakes detached from the substrate, exposing the graphite layer. On the contrary, for GS/NiFe alloy/NiFe LDH electrode, the NiFe LDH layer remained closely attached to the substrate despite some cracks being present. The surface composition and chemical bonding states of the GS/NiFe LDH, GS/NiFe alloy, and GS/NiFe alloy/NiFe LDH films were analyzed by XPS. The high-resolution XPS spectra of Ni *2p*, Fe *2p*, and P *2p* regions are presented in ***Supplementary Figs. 14*** and ***15***; all pristine films exhibited signals characteristic of Ni^{2+} and Fe^{3+}. In the GS/NiFe LDH film, the peak intensity diminished after the oxygen evolution reaction (OER) test due to the detachment of the NiFe LDH from the substrate, which was consistent with SEM results. In the GS/NiFe alloy film, the small Ni *2p* peak at about 852 eV was attributed to metallic nickel species in the alloy, while the small Fe *2p* peak at about 706 eV was attributed to metallic iron species in the alloy. The presence of Ni^{2+} and Fe^{3+} peaks was due to the surface oxidation of the NiFe alloy. The NiFe alloy should contain phosphide components due to the use of phosphate in the plating bath. In the P *2p* area, peaks with binding energies of 133 eV corresponded to the central phosphorus atoms in phosphate species. The absence of a signal of metal phosphide (129 eV) was due to the surface oxidation of the NiFeP alloy. After the OER catalysis, the Fe^0, Ni^0, and phosphate signals diminished, suggesting the surface oxidation or surface reconstruction of the alloy to (hydro)oxide species. In the GS/NiFe alloy/NiFe LDH film, no metallic iron, metallic nickel, or phosphate species were detected, implying that the alloy layer is fully covered by the LDH layer.

All the spectra showed no noticeable change after the OER test, further confirming the chemical stability of the NiFe LDH catalyst.

The XRD analysis of the catalyst films was carried out to further investigate the structural evolution during catalysis. As shown in ***Supplementary Fig. 16a***, there is no discernible difference between the pristine films of GS and GS/NiFe LDH, indicating that the NiFe LDH layer is amorphous. The presence of Ni-base alloy is confirmed by the characteristic peaks of Ni (111) and (200) in both GS/NiFe alloy and GS/NiFe alloy/NiFe LDH. After the OER, additional peaks that may be attributed to α-$Ni(OH)_2$ are detected in both GS/NiFe alloy and NiFe LDH electrodes, suggesting a similar surface structural reconstruction process occurred for both NiFe alloy and NiFe LDH, leading to the generation of highly active NiFe species (***Supplementary Fig. 16b***). The XPS and XRD results suggest that surface structural reconstruction may have occurred on the NiFe surface and also confirm the composition stability of the NiFe LDH component after water oxidation catalysis.

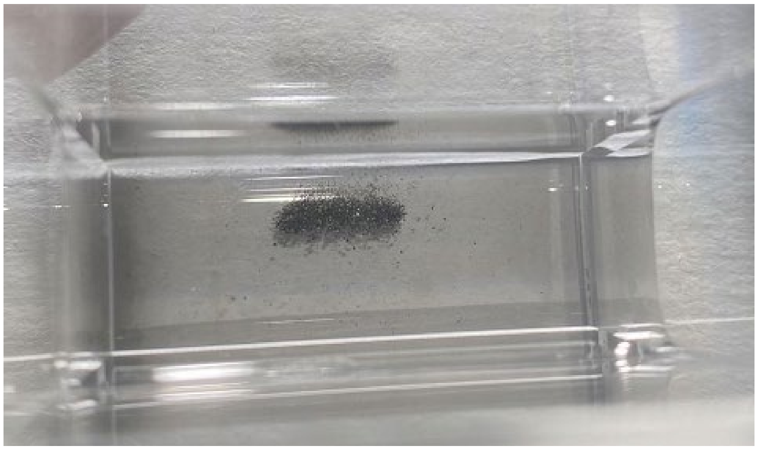

Supplementary Fig. 10 Digital image of precipitates from GS/NiFe LDH electrode in electrolyzer.

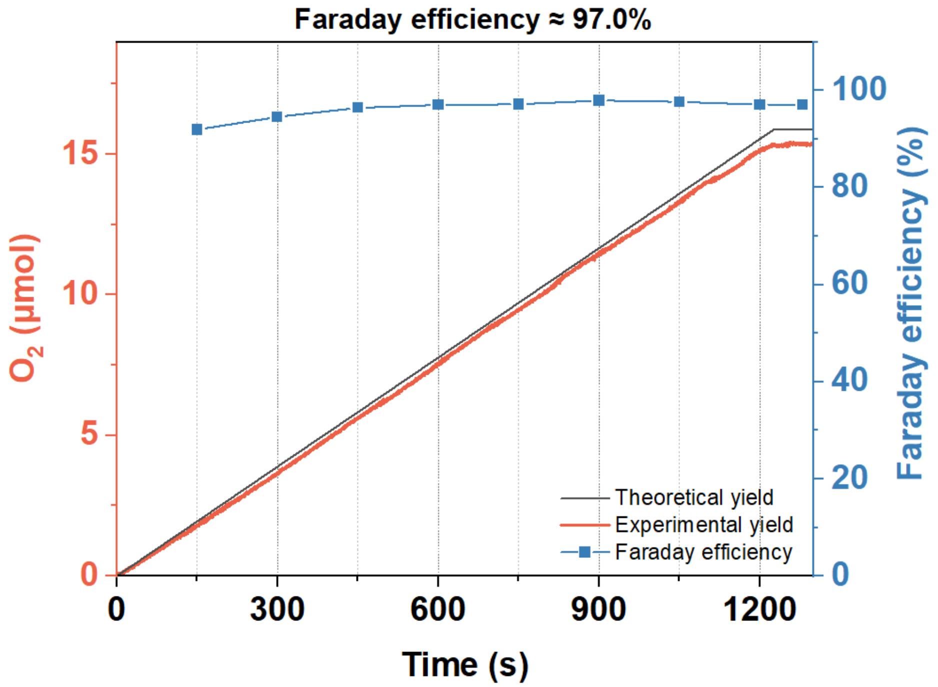

Supplementary Fig. 11 Faradaic efficiencies of GS/NiFe alloy/NiFe LDH for the OER in 1.0 M KOH. Theoretically calculated and experimentally measured amounts of O_2 were shown as functions of electric charge for water oxidation catalyzed by GS/NiFe alloy/NiFe LDH at a current density of 5 mA cm^{-2} (electrode area = 1.0 cm^2).

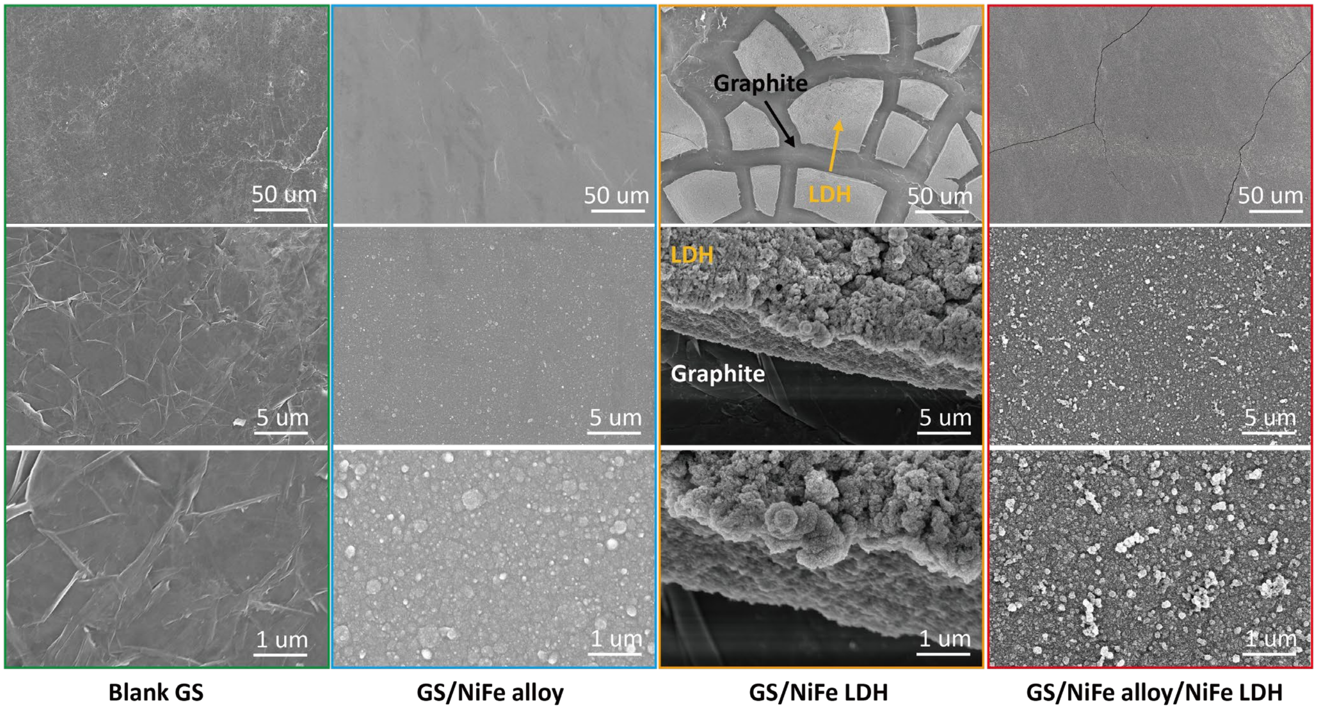

Supplementary Fig. 12 SEM images of blank GS, GS/NiFe alloy/NiFe LDH, GS/NiFe alloy, and GS/NiFe LDH under different magnifications.

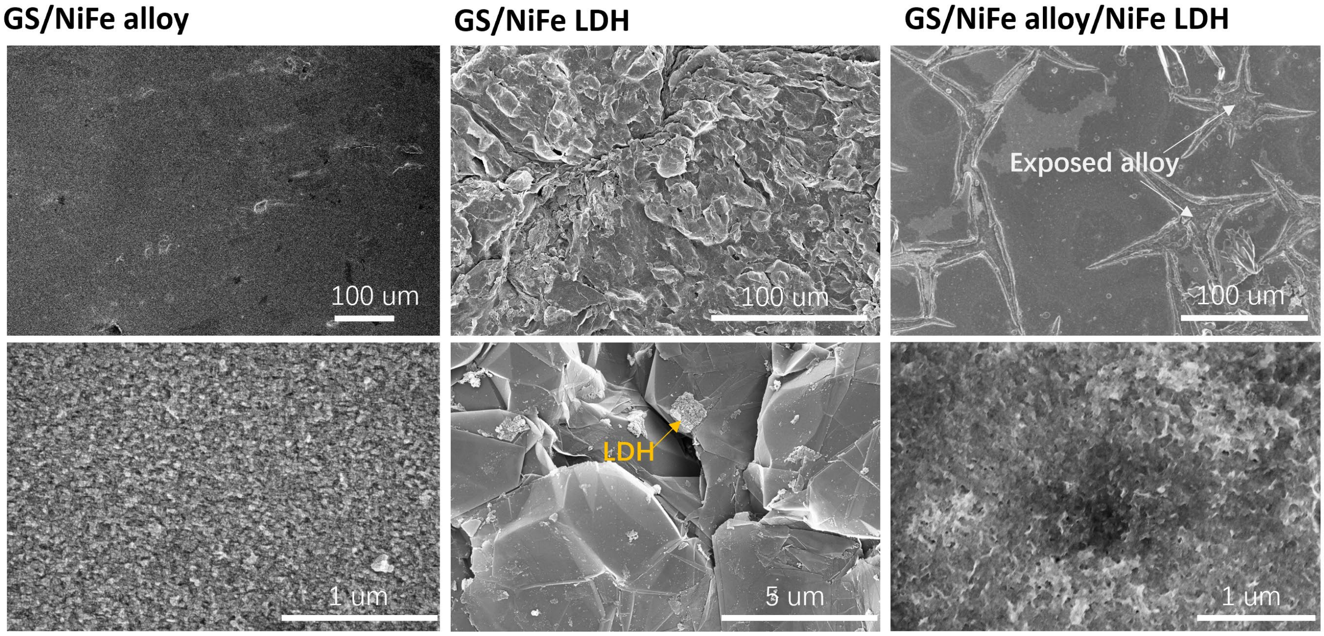

Supplementary Fig. 13 SEM images of blank GS, GS/NiFe alloy/NiFe LDH, GS/NiFe alloy, and GS/NiFe LDH after 120 hours' electrolysis at a constant current density of 10 mA cm^{-2}.

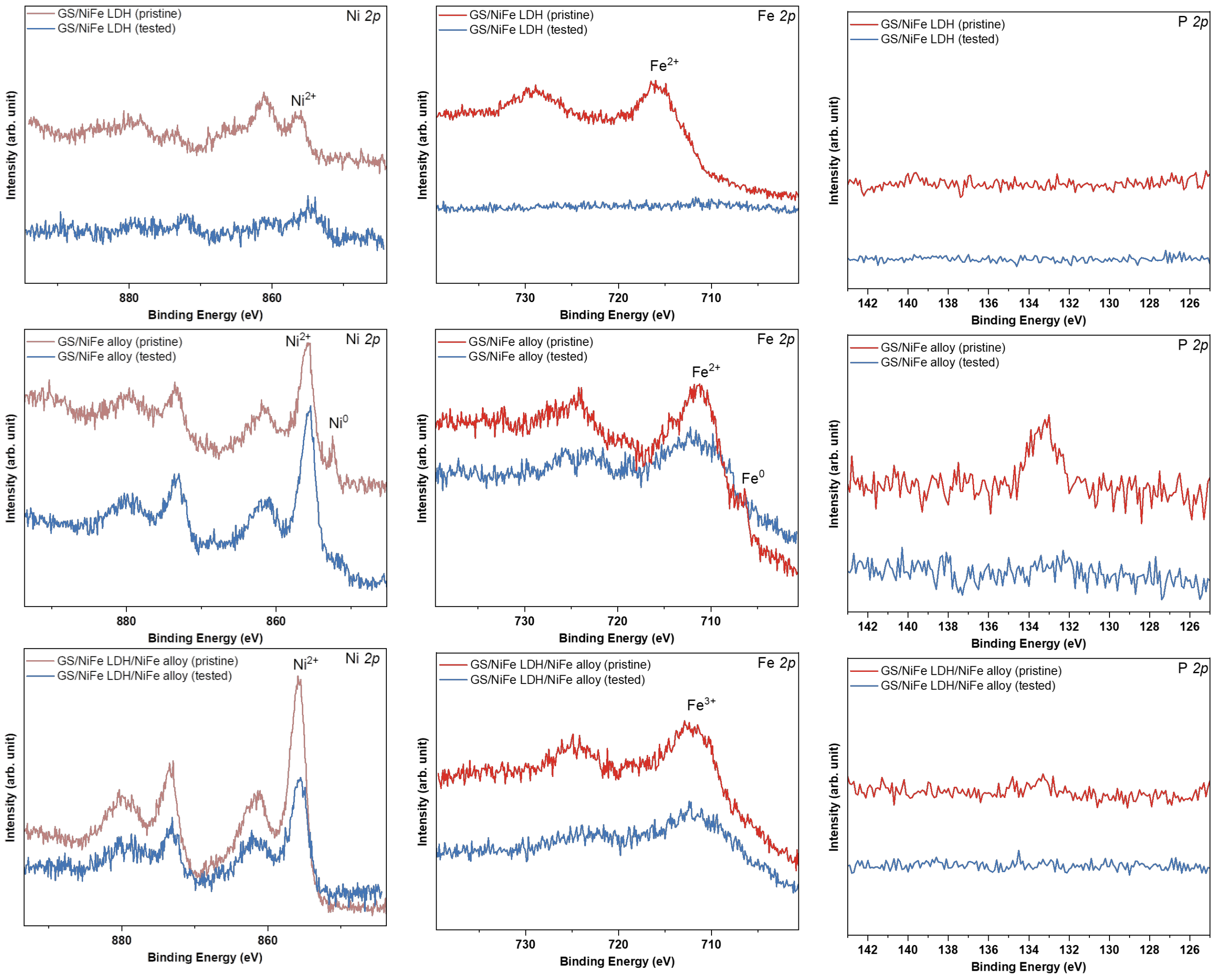

Supplementary Fig. 14 High resolution-XPS spectra of the as-prepared electrode in selective Ni *2p*, Fe *2p*, and P *2p* areas.

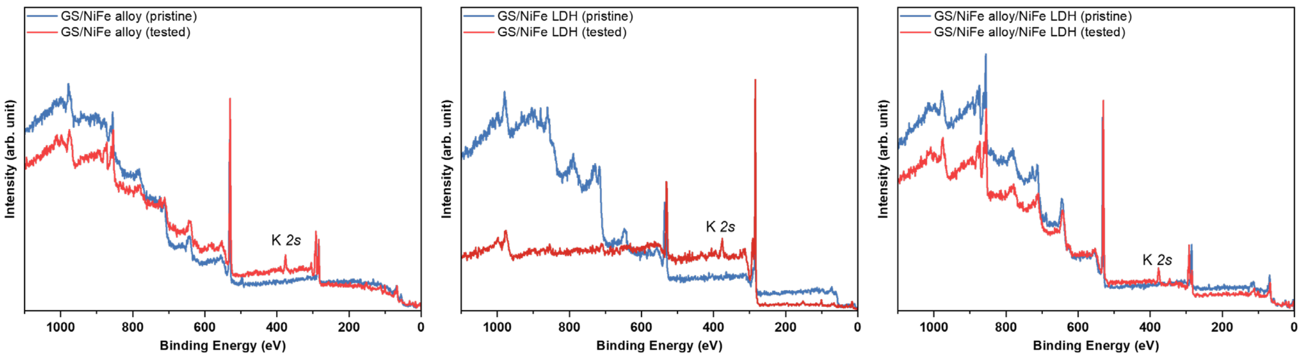

Supplementary Fig. 15 XPS survey spectra of the as-prepared electrode.

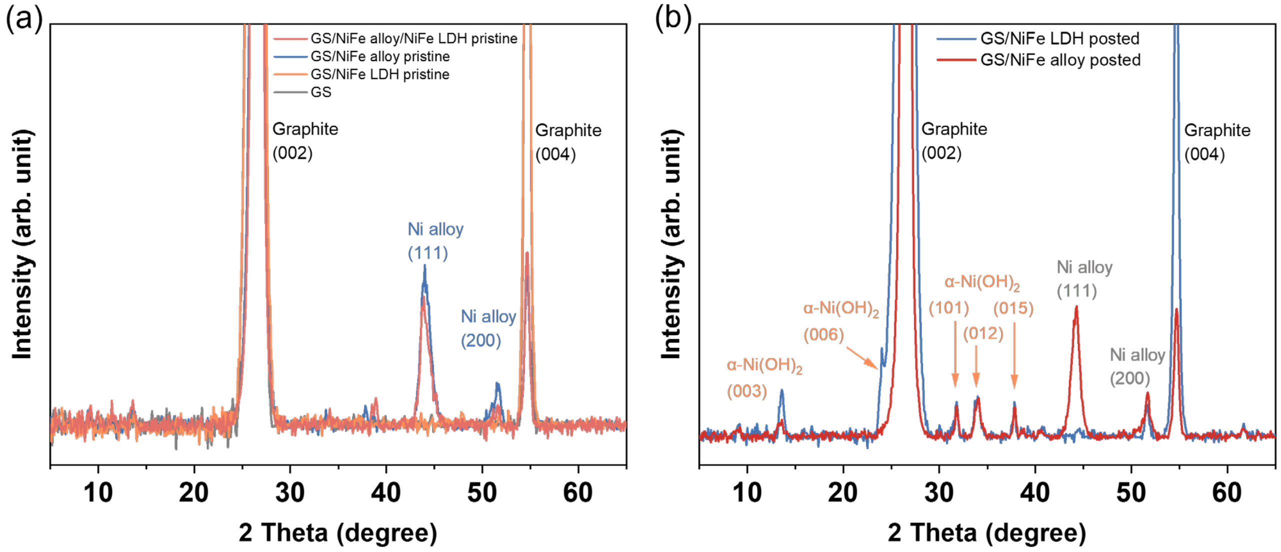

Supplementary Fig. 16 XRD spectra of (a) pristine and (b) test samples.

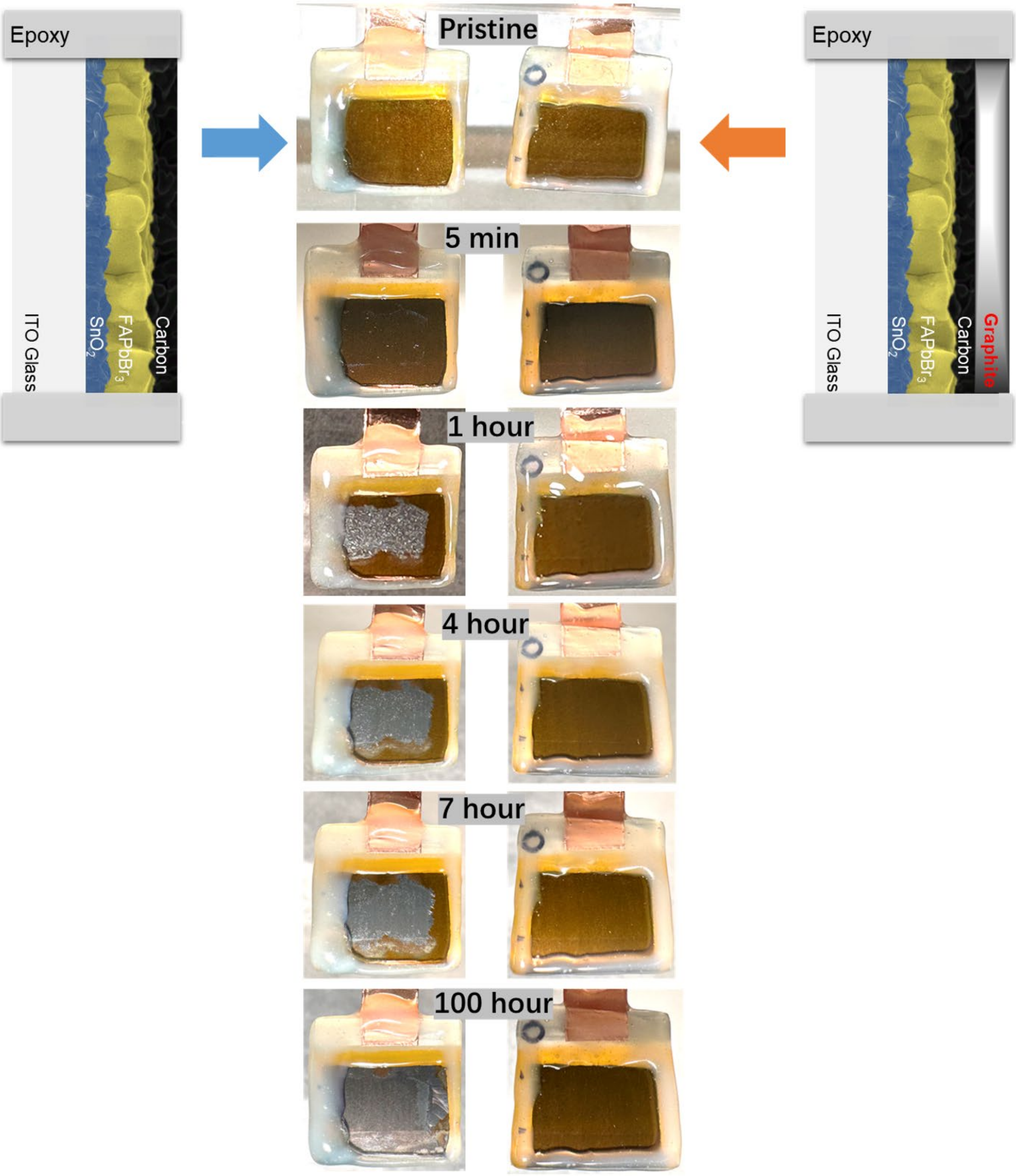

Supplementary Fig. 17 Waterproof performance test of carbon-protected and GS-protected devices. The devices were immersed in pure water for 100 hours.

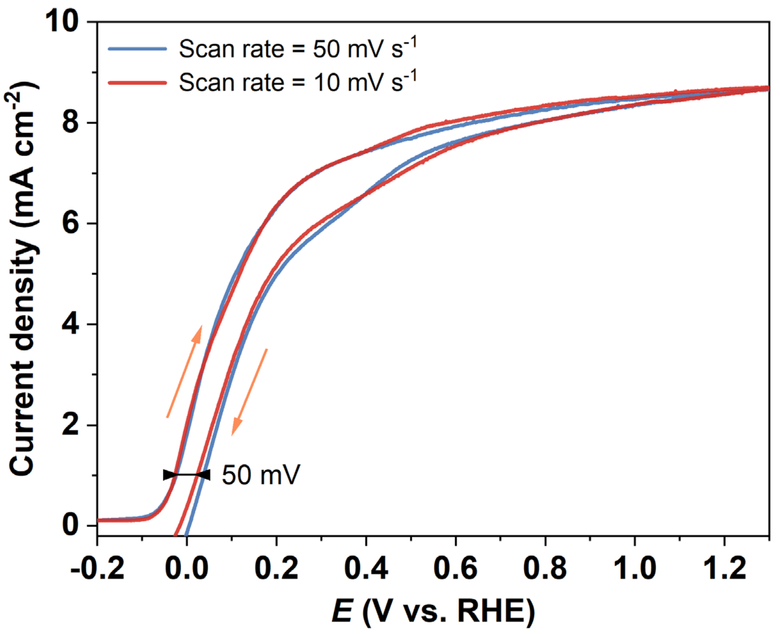

Supplementary Fig. 18 Cyclic voltammetry (CV) curves of $FAPbBr_3$ photoanode with a scan rate of 50 mV s^{-1} and 10 mV s^{-1} in 1.0 M KOH.

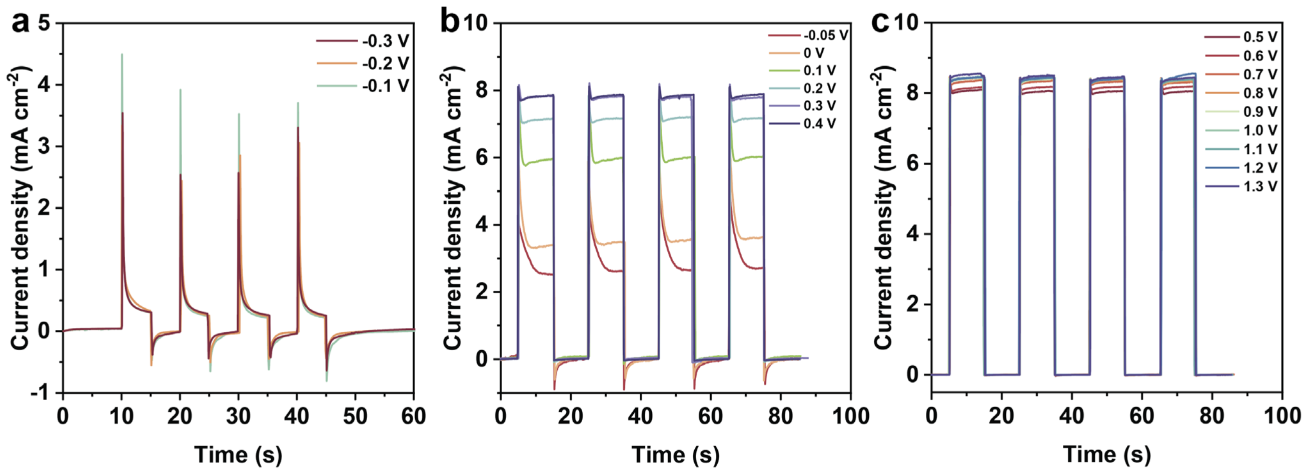

Supplementary Fig. 19 (a-c) light response of $FAPbBr_3$ photoanode under chopped irradiation at a constant bias of −0.3 to 1.3 V vs. reversible hydrogen electrode (RHE).

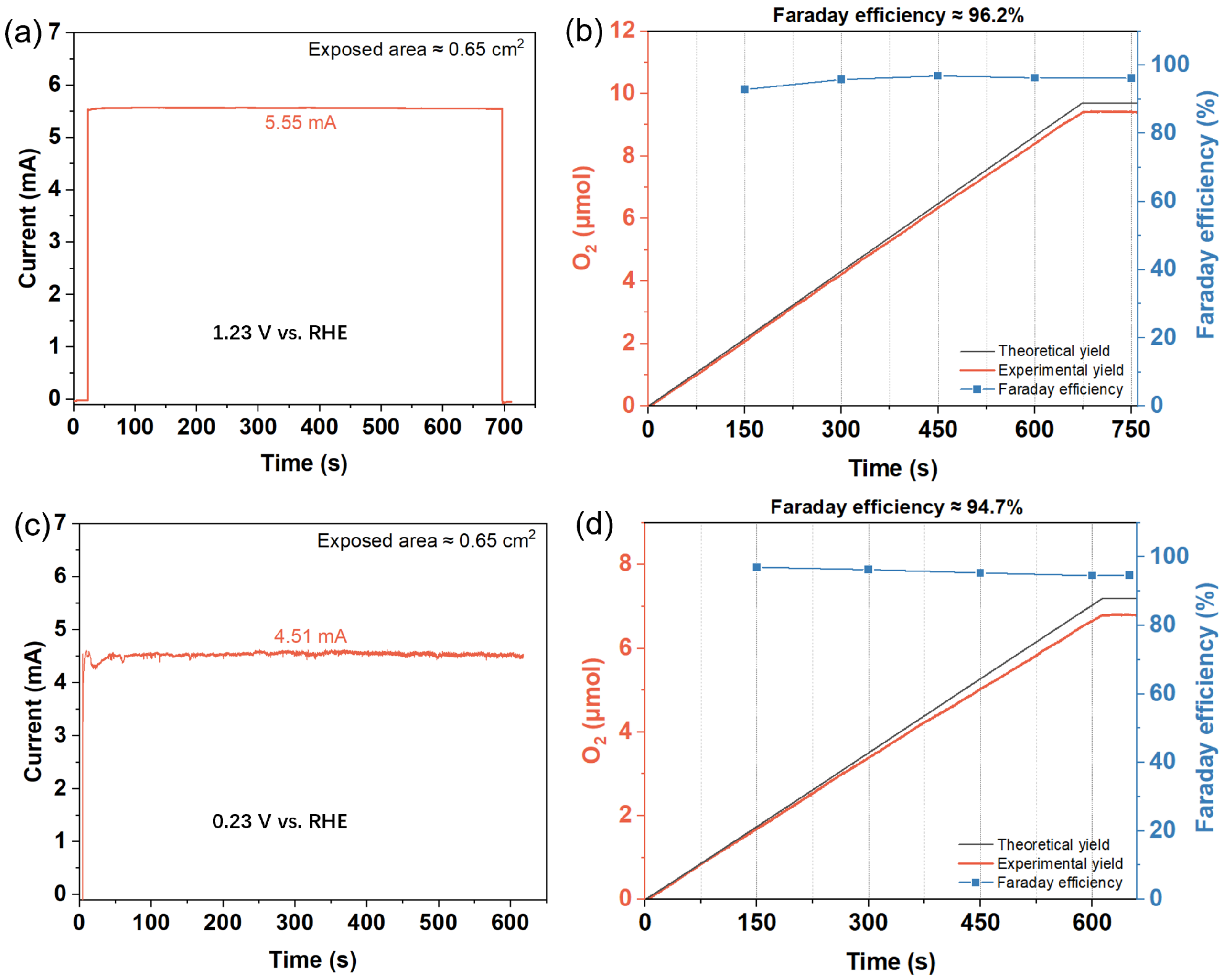

Supplementary Fig. 20 The current of $FAPbBr_3$ photoanode at a constant potential of (a) 1.23 V and (c) 0.23 V vs. RHE during Faradaic efficiency measurements. Faradaic efficiencies of $FAPbBr_3$ photoanode for the light-driven OER in 1.0 M KOH. Theoretically calculated and experimentally measured amounts of O_2 were shown as functions of electric charge for water oxidation at a constant potential of (b) 1.23 V and (d) 0.23 V vs. RHE (electrode area = 0.65 cm^2).

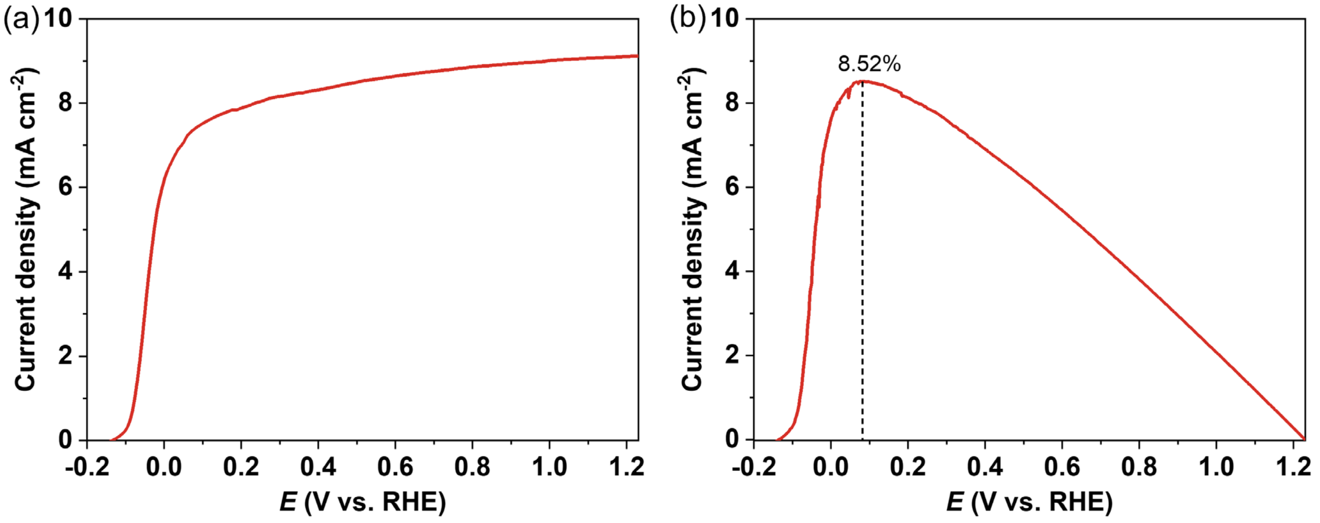

Supplementary Fig. 21 (a) LSV curve of $FAPbBr_3$ photoanode in 1.0 M KOH (scan rate: 50 mV s^{-1}). (b) corresponding applied bias photon-to-current efficiency (ABPE) calculated from the LSV curve.

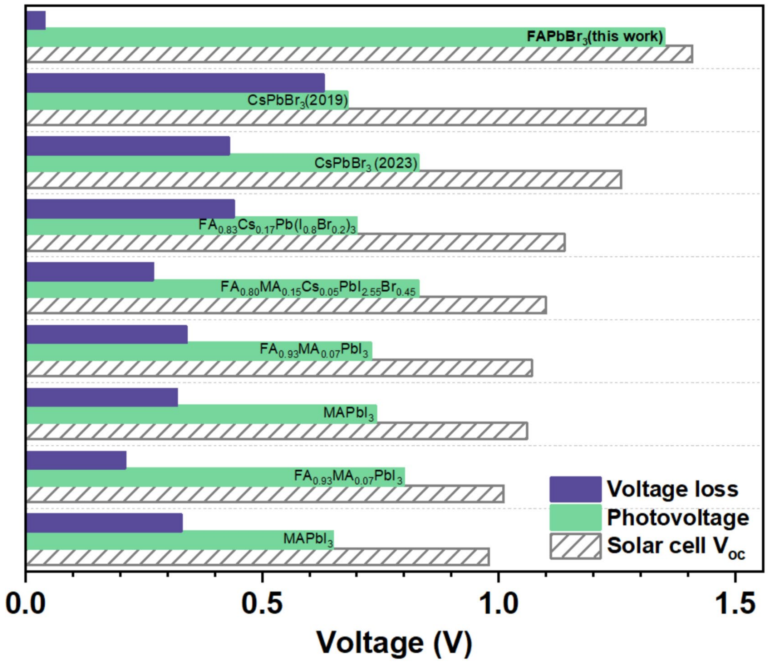

Supplementary Fig. 22 Comparison of solar cell V_{oc}, photoanode photovoltage, and voltage loss of the reported perovskite photoanodes for water oxidation. Voltage loss is defined as the difference between solar cell V_{oc} and photoanode photovoltage (**Table S5**).

Supplementary Table 4 ABPEs of the reported representative photoanodes for water oxidation.

Device	Maximum ABPE (%)	Potential (V vs. RHE)	Ref.
np$^+$-Si/SiO$_x$/NiO$_x$/NiFe	3.3	1.08	[10]
Polycrystalline n$^+$p-Si/NiFe-LDH	4.3	1.02	[11]
n-Si/SiO$_x$/Al$_2$O$_3$/Ni/NiO$_x$/NiOOH	3.0	1.05	[12]
NiFe/SiO$_x$/n-Si	3.12	1.09	[13]
n-Si/Ni/NiOOH/NiFe	1.42	1.16	[14]
NiFeP/n-Si	0.37	1.18	[15]
NiOOH/FeOOH/Co$_3$O$_4$/BiVO$_4$	2.72	0.60	[16]
Co@CB[5]/BiVO$_4$	1.79	0.60	[17]
BiVO$_4$/NiFeO$_x$	2.76	0.58	[18]
CoOOH/g-C$_3$N$_4$/BiVO$_4$	1.65	0.62	[19]
FeOOH/Ni-N$_4$-O/BiVO$_4$	2.66	0.54	[20]
Co$_4$O$_4$ cubane/BiVO$_4$	1.84	0.70	[21]
FA$_{0.93}$MA$_{0.07}$PbI$_3$/NF.3D Ni.NiFe	9.16	0.75	[2]
FA$_{0.83}$Cs$_{0.17}$Pb(I$_{0.8}$Br$_{0.2}$)$_3$/Ni	5.60*	0.83*	[3]
FA$_{0.80}$MA$_{0.15}$Cs$_{0.05}$PbI$_{2.55}$Br$_{0.45}$/FeNi(OH)$_x$	4.25*	0.77*	[22]
MAPbI$_3$/Ni	3.86*	0.85*	[23]
MAPbI$_3$/Ni	3.20*	0.91*	[24]
CsPbBr$_3$/Ir WOC	1.02*	0.87*	[1]
CsPbBr$_3$/NiFeOOH	3.30*	0.67*	[25]
(5-AVA)$_x$(MA)$_{1-x}$PbI$_3$/Carbon	0.88	1.08	[26]
FTO/mZnO/BHJ/PTAA/LIO	0.56	0.76*	[27]
FAPbBr$_3$ photoanode	8.52	0.082	This work

*Data extract from the LSV curve.

Supplementary Table 5 Photovoltage of the reported perovskite photoanodes for water oxidation.

Perovskite composition	Bandgap (eV)	Photovoltage (V)	Solar cell V_{oc} (V)	Ref.
$(5\text{-AVA})_x(MA)_{1-x}PbI_3$#	1.5	0.29	-	[26]
$FA_{0.83}Cs_{0.17}Pb(I_{0.8}Br_{0.2})_3$	1.66	0.7	1.14	[3]
$MAPbI_3$	1.5	0.74	1.06	[23]
$FA_{0.93}MA_{0.07}PbI_3$	1.55	0.73	1.07	[2]
$CsPbBr_3$ (2019)#	2.3	0.68	1.31	[1]
$CsPbBr_3$ (2023)#	2.3	0.83	1.26	[25]
$FA_{0.93}MA_{0.07}PbI_3$	1.55	0.8	1.01	[28]
$FA_{0.80}MA_{0.15}Cs_{0.05}PbI_{2.55}Br_{0.45}$	1.57	0.83	1.1	[22]
$MAPbI_3$	1.5	0.65	0.98	[24]
$FAPbBr_3$#	2.25	1.35*	1.4	This work

HTM-free device.

*Data extract from the device with best ABPE.

Supplementary Table 6 Photovoltage of the reported polymer bulk heterojunction (BHJ) photoanode for water oxidation.

Composition	Bandgap (eV)	Photovoltage (V)	Solar cell V_{oc} (V)	Ref.
PNDITCVT/PBDTTTPD	2.04*	1.03	-	[27]

*Bandgap values are estimated from the difference between the lowest LUMO and highest HOMO of PNDITCVT/PBDTTTPD junction.

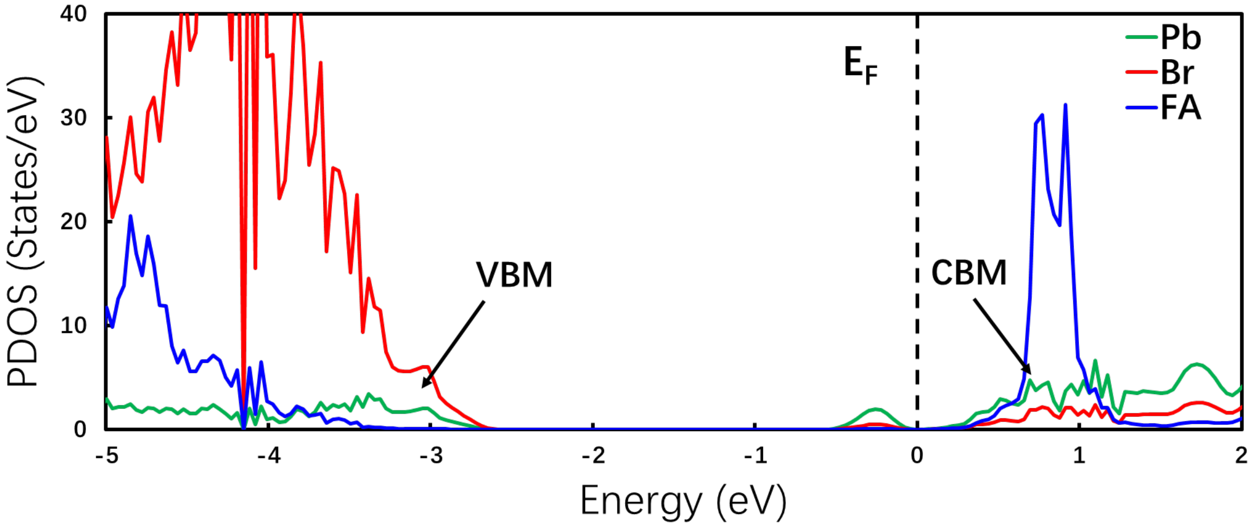

Supplementary Fig. 23 Calculated partial density of states (PDOS) for X-site vacancy (V_{Br}).

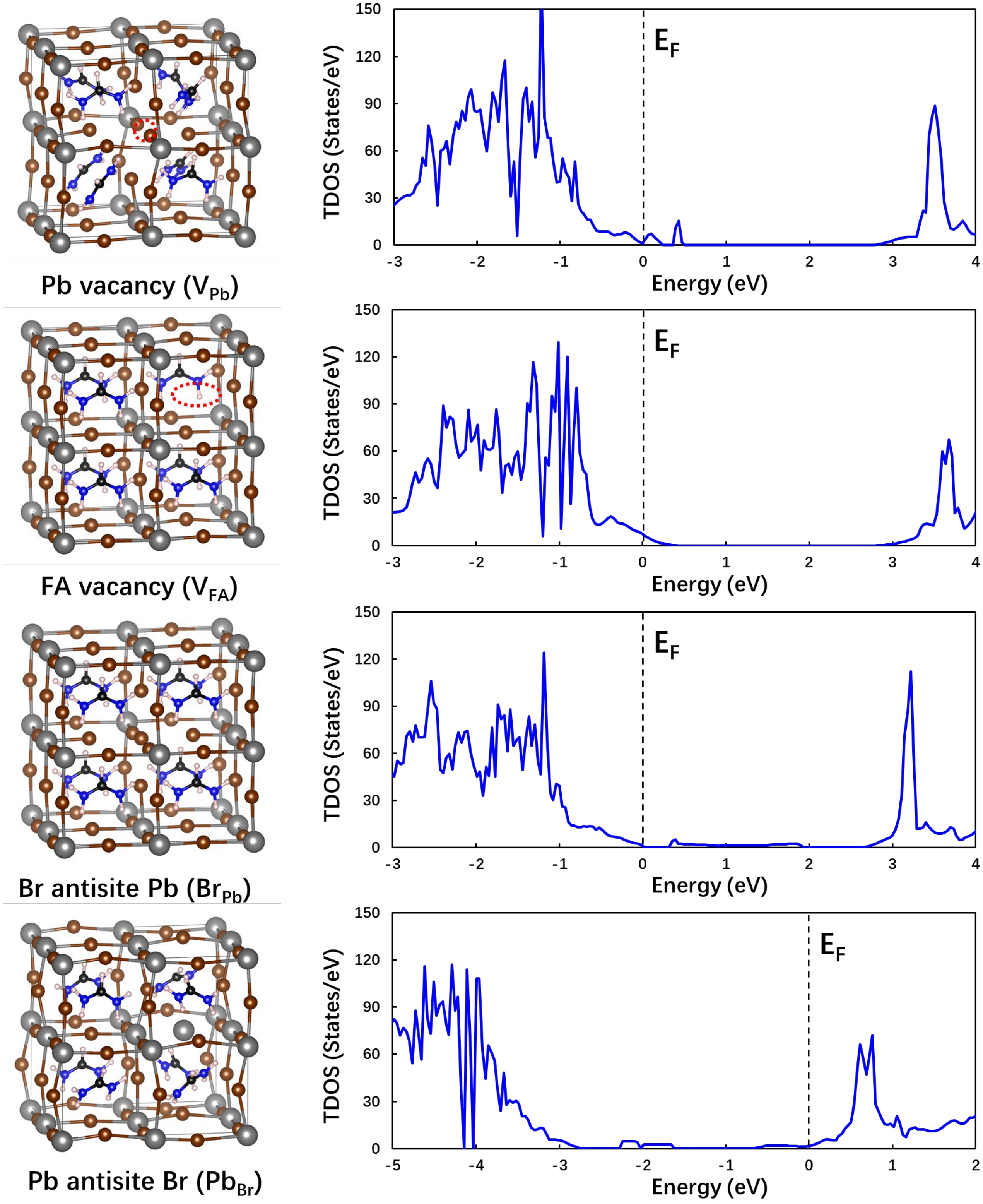

Supplementary Fig. 24 Geometric structures and calculated total density of states of defective $FAPbBr_3$ perovskites.

Supplementary Note 3: Calculation for defect formation energy.

For a defect D with a charge-state q, defect formation energy (DFE, ΔH_f) over the slab surface is calculated from the following ***Supplementary Equation 1***:[29]

$$\Delta H_f(\mathrm{D},q) = E_{def}(\mathrm{D},q) - E_{pristine} - \sum_i n_i \mu_i + q(E_F + E_{VBM}) \quad (1)$$

where $E_{pristine}$ is the total energy of the defect-free slab, μ_i are the chemical potentials, n_i are the change in the number of atoms during the formation of the defect, E_F is the Fermi energy and E_{VBM} is the valence band maximum (VBM) energy. The chemical potential, for the formation of $FAPbBr_3$, should satisfy

$$\mu_{FA} + \mu_{Pb} + 3\mu_{Br} = \mu_{FAPbBr_3} \quad (2)$$

under thermodynamic equilibrium growth conditions, where $\mu_{FAPbBr3}$ is the formation enthalpy of $FAPbBr_3$. In addition, due to the experimental synthesis methods, we suppose it is in equilibrium with the $PbBr_2$ phase. Moreover, the FABr phase is not supposed to form. Thus, the ***Supplementary Equations 3*** and ***4*** below should be satisfied:

$$\mu_{Pb} + 2\mu_{Br} = \mu_{PbBr_2} \quad (3)$$

$$\mu_{FA} + \mu_{Br} < \mu_{FABr} \quad (4)$$

When the Pb-rich is considered (depending on the fabrication and other conditions), $\mu_{Pb} = \mu_{Pb,bulk}$ can be determined, where $\mu_{Pb,bulk}$ is the energy of a Pb atom in bulk Pb metal. Then $\mu_{Br} = (\mu_{PbBr2} - \mu_{Pb})/2$ can be met.

Supplementary Table 7 presents the calculated DFEs for Pb_{Br}, V_{Br}, Br_{Pb}, and V_{Pb} as 0.78, -0.51, 3.49, and 0.86 eV, respectively. Consequently, under Pb-rich conditions, the $FAPbBr_3$ system with a Br vacancy exhibits the lowest DFE and, thus, possesses the most stable structure.

Supplementary Table 7 Formation energies of neutral defects considered in this work. The energies are in eV.

Defect type	ΔH_f (eV)
Pb_{Br}	0.78
V_{Br}	-0.51
Br_{Pb}	3.49
V_{Pb}	0.86

Supplementary Note 4: Flatband potential and onset potential analysis.

The flat-band potential (E_{fb}) of a semiconductor-electrolyte interface is the potential at which the energy bands of the semiconductor are flat at the interface, and there is no net movement of charge across the interface. When the potential applied to the photoanode is more cathodic than the flat-band potential, the transfer of photogenerated holes toward the semiconductor/electrolyte interface is forbidden due to the Schottky barrier. When the potential applied to the photoanode is more anodic than the flat-band potential, photogenerated holes will be attracted towards the semiconductor/electrolyte interface due to the downward band bending, where they can be possible to react with water molecules and contribute to the water oxidation reaction. As shown in ***Supplementary Fig. 25a***, if the quasi-Fermi energy level of the holes (${}_{p}E_{F}$) is shallower than the thermodynamic potential required for water oxidation (E_{OER}), the water oxidation reaction still cannot occur due to the smaller driven force. As a result, the onset potential (E_{onset}) is more anodic than E_{fb}. On the other hand, when ${}_{p}E_{F}$ is deeper than E_{OER}, OER will immediately occur when the applied potential exceeds E_{fb} (***Supplementary Fig. 25b***). For specific semiconductor materials, there are mainly two methods to lower the onset potential; one is to increase the V_{ph} of the material through specific modification, and the other is to use a more efficient catalyst to reduce the overpotential of E_{OER}.

For a diode, the flat band potential is actually the difference between the Fermi levels of the two materials. Similarly, in the semiconductor/electrolyte system, when normal hydrogen electrode (NHE) or RHE is used as the potential reference, the flat band potential is the NHE/RHE potential corresponding to the Fermi level of the semiconductor in vacuum. Even for heavily doped n-type semiconductors, the lowest Fermi level is considered to be the edge of the conduction band (with "high" and "low" being relative to RHE/NHE rather than the vacuum level). Since the energy band level is an intrinsic property of the semiconductor given a certain fabrication procedure, the minimum E_{onset} of the photoanode will not be shallower than the conduction band minimum (CBM) of the semiconductor material. Therefore, in order to prepare a photoanode with a lower E_{onset}, the CBM of the semiconductor material should be lowered (i.e., higher value relative to the vacuum level) while ensuring sufficient photoelectrochemical voltage.

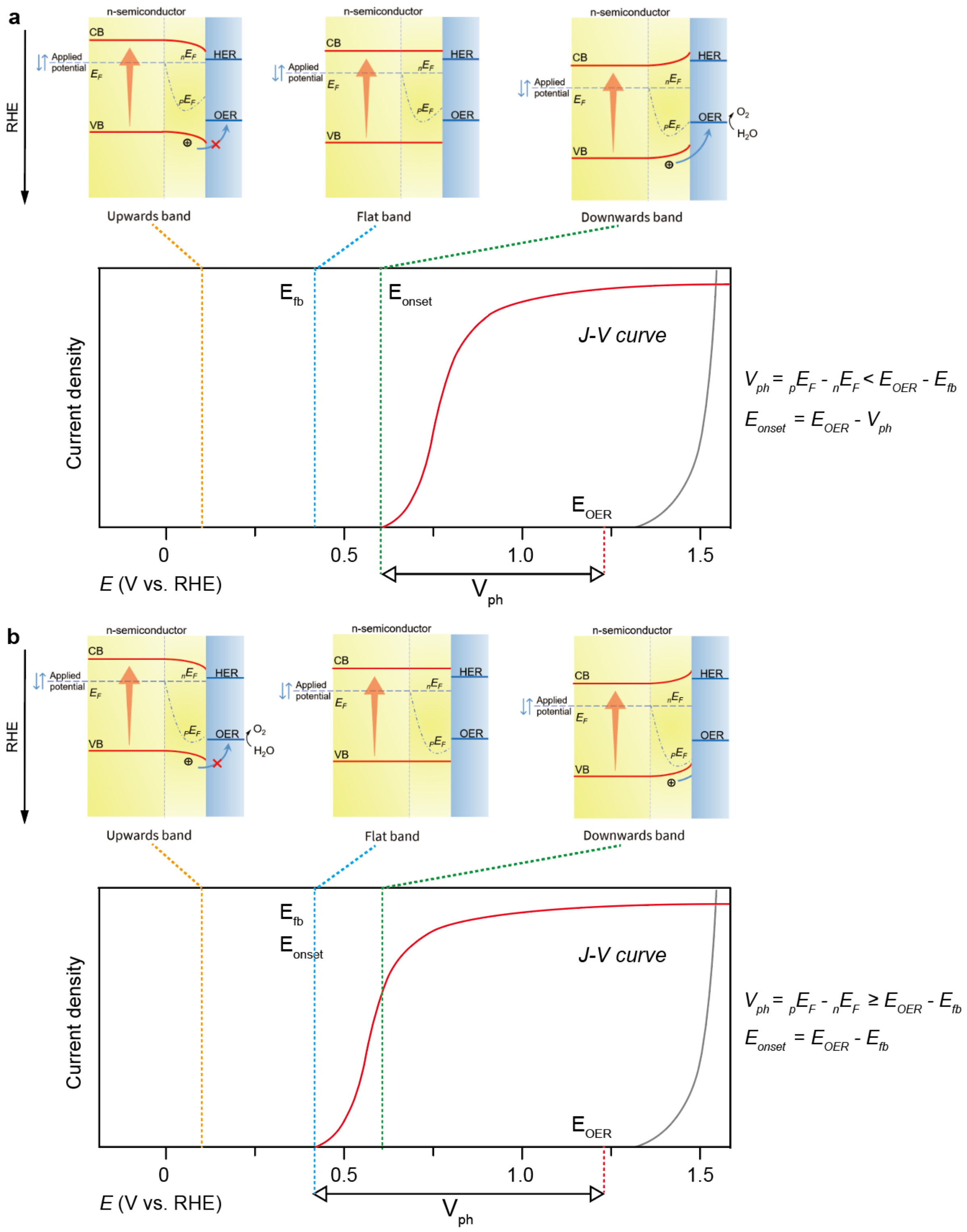

Supplementary Fig. 25 Energy diagram and schematic *J-V* curve for n-type semiconductor-based photoanode in contact with an aqueous electrolyte under different applied potentials: (a) V_{ph} is smaller than the difference between E_{OER} and E_{fb}. (b) V_{ph} is larger than the difference between E_{OER} and E_{fb}.

Supplementary Fig. 26a shows the energy band positions of commonly studied oxide and perovskite semiconductors. It is evident that the CBM of $FAPbBr_3$ is significantly shallower than those of the other candidates; UPS results show that the Fermi level of the $FAPbBr_3$ film is -0.7 V vs. NHE (−3.7 eV vs. vacuum). We assume that the Fermi level of the oxide semiconductor is very close to the CBM. Therefore, the order of the flat band potential should be $FAPbBr_3$ < TiO_2 < $BiVO_4$ < Fe_2O_3. Assuming that these high-performance wide-bandgap photoanodes are capable of driving water oxidation reactions at flat-band potentials ($V_{ph} > E_{oer} - E_{fb}$), thus the order of their theoretical onset potentials should also be $FAPbBr_3$ < TiO_2 < $BiVO_4$ < Fe_2O_3. The practical experimental E_{onset} in ***Supplementary Fig. 26b*** confirmed the above speculation. Moreover, the Fermi level of $FAPbBr_3$ is shallower than the CBM of $MAPbI_3$ and $FAPbI_3$, which also accounts for the origin of the ultralow onset potential of the $FAPbBr_3$ photoanode when compared to other perovskite photoanodes. In summary, the shallower Fermi level and higher photovoltage result in the ultralow onset potential of the $FAPbBr_3$ photoanode.

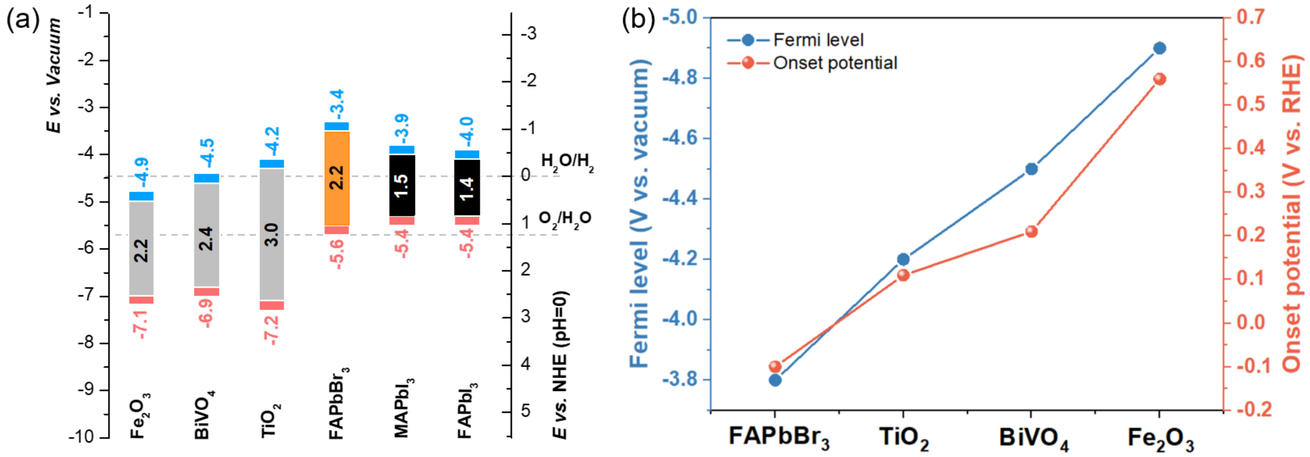

Supplementary Fig. 26 (a) Band positions of Fe_2O_3, $BiVO_4$, TiO_2, $FAPbBr_3$, $MAPbI_3$, and $FAPbI_3$ in the pH 0 aqueous electrolyte compared with the energy potential for water splitting reaction. (b) The potential of VBM and E_{onset} of the best-reported Fe_2O_3,[30] $BiVO_4$[31] TiO_2,[32] and $FAPbBr_3$ photoanodes.

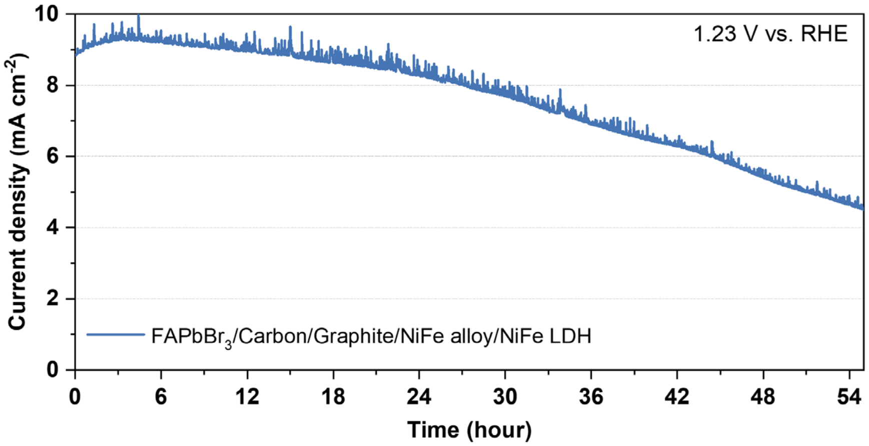

Supplementary Fig. 27 Chronoamperometric measurement of $FAPbBr_3$ photoanode at 1.23 V vs. RHE in 1.0 M KOH solutions (AM 1.5G, 100 mW cm^{-2}).

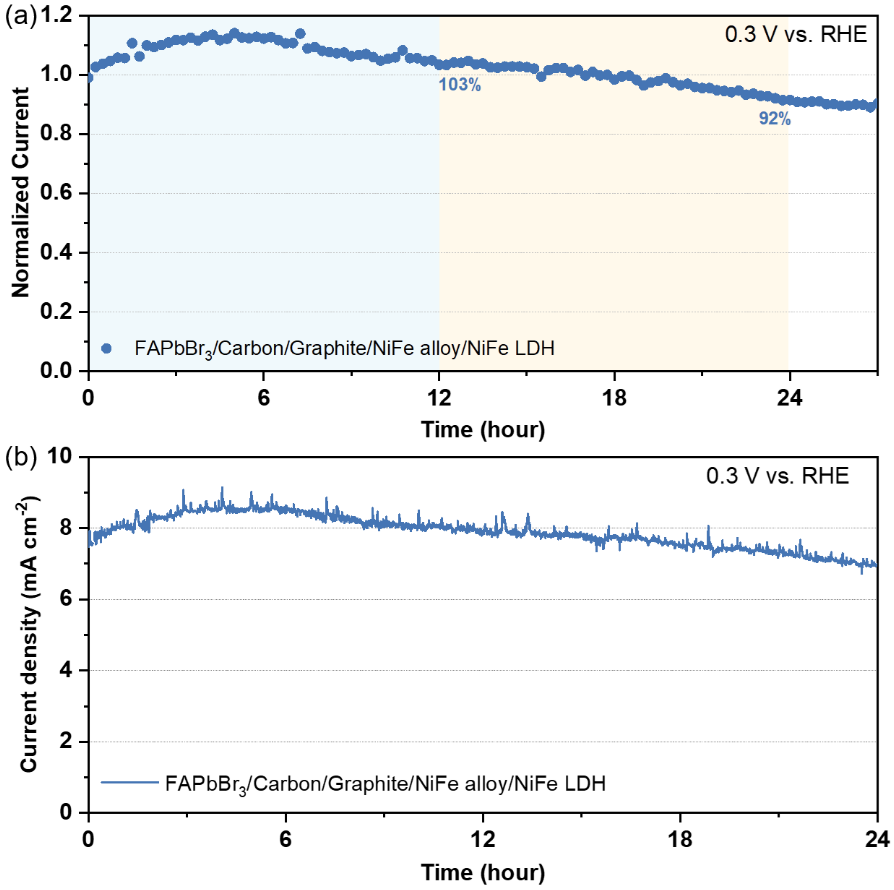

Supplementary Fig. 28 Chronoamperometric measurement of $FAPbBr_3$ photoanode at 0.3 V vs. RHE in 1.0 M KOH solutions (AM 1.5G, 100 mW cm^{-2}). (a) Normalized current and (b) apparent current density.

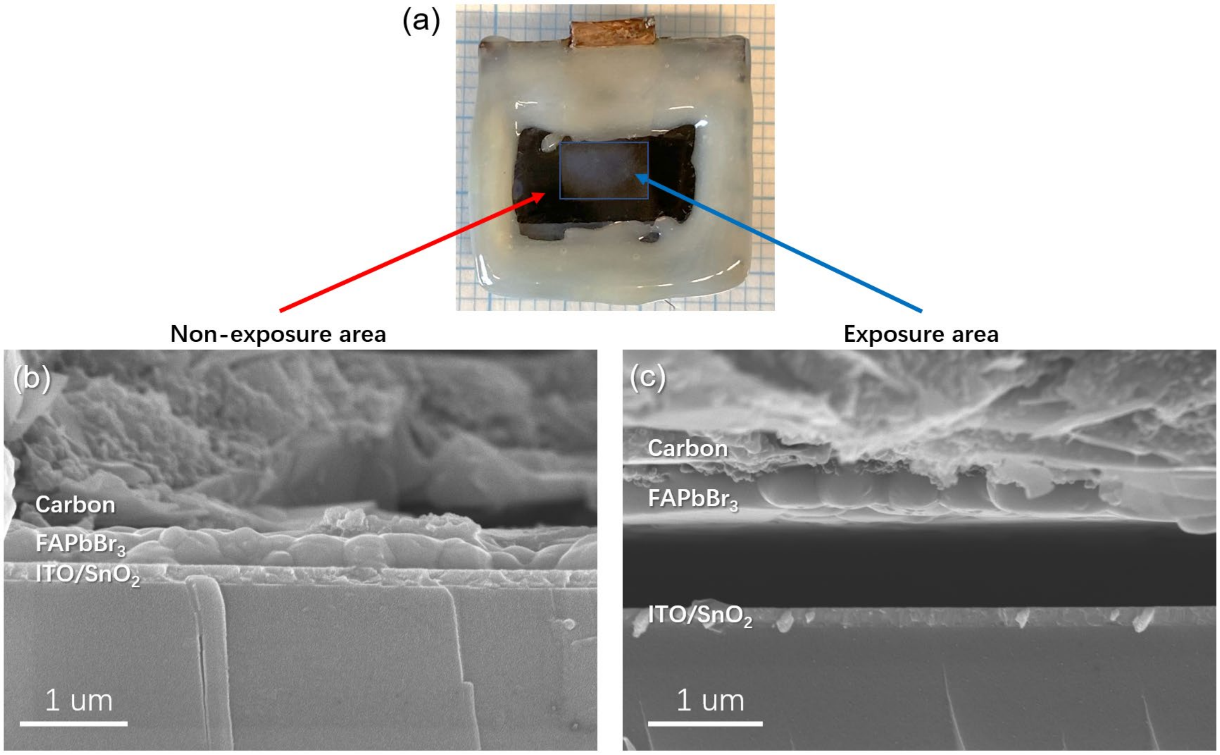

Supplementary Fig. 29 (a) Digital image of selectively exposed photoanode after over 50-hours' stability test. SEM images for the (b) non-exposed and (c) exposed parts of the tested photoanode in cross-section view.

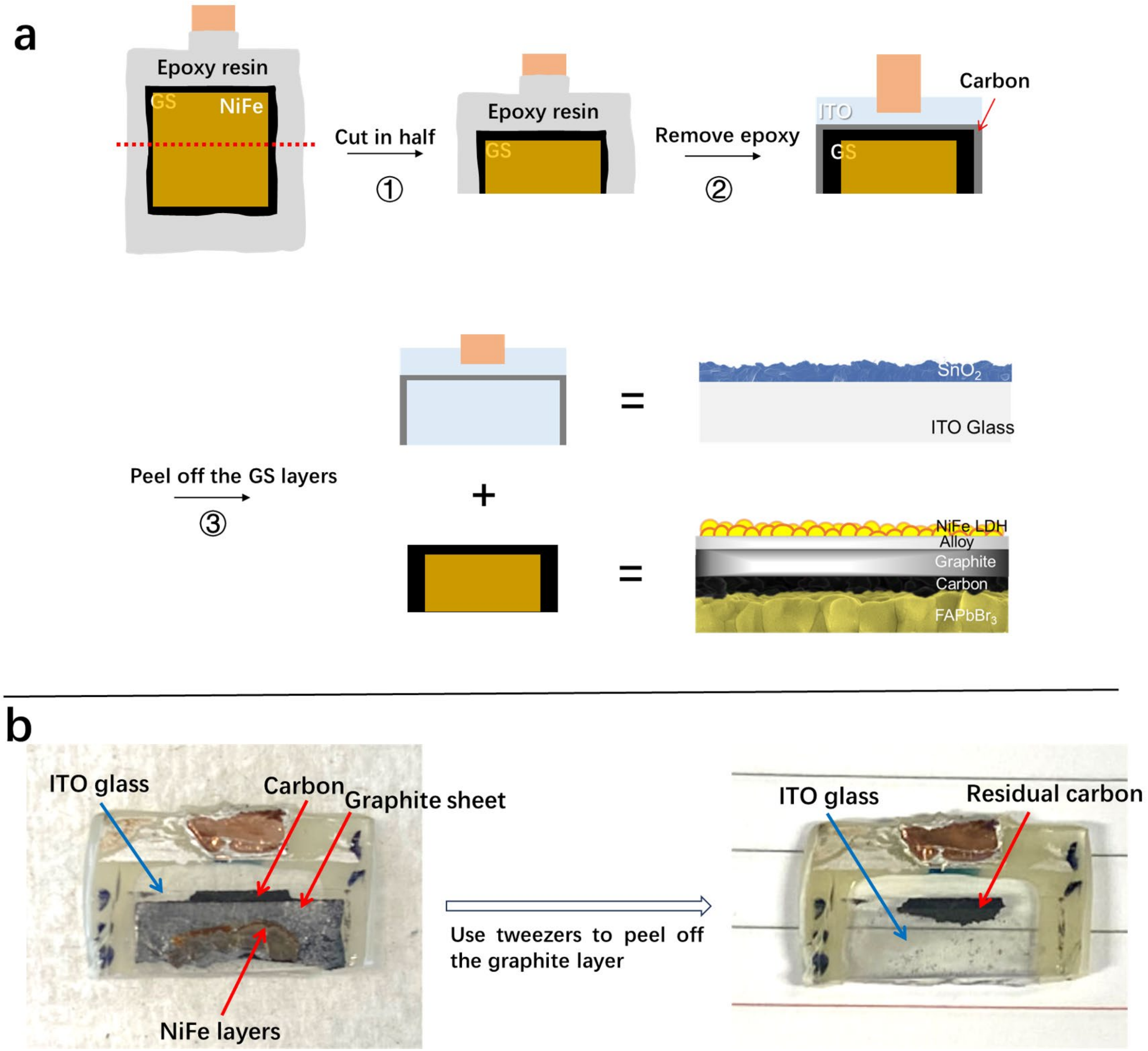

Supplementary Fig. 30 (a) Schematic diagram of disassembling the tested photoanode. (b) Digital images of disassembled photoanode. When an external force is applied to remove the surrounding resin glue (step 2), a distinctive peeling effect is observed at the interface between the glass/SnO_2 layers and the $FAPbBr_3$/carbon/graphite sheet layers, which facilitates the easy separation of the graphite sheet from the glass substrate (step 3). This phenomenon is specifically attributed to the degradation occurring at the SnO_2 interface.

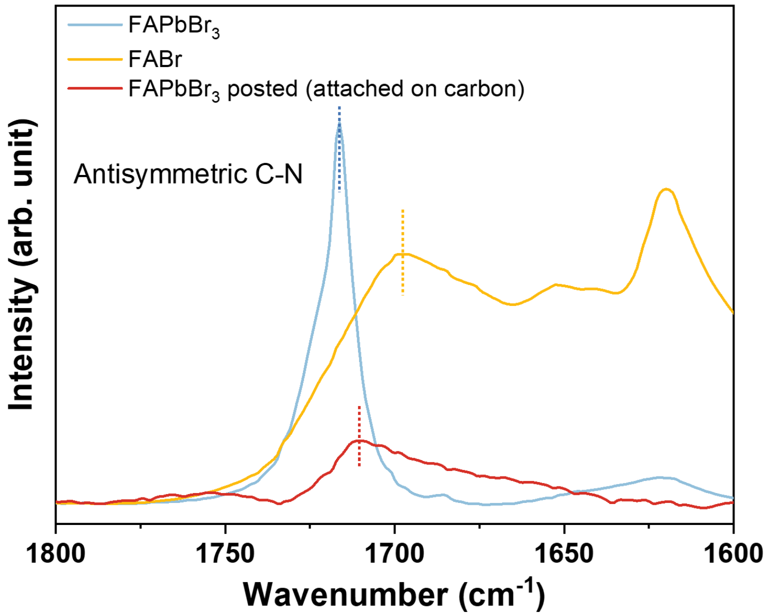

Supplementary Fig. 31 FT-IR spectra of the pristine and tested $FAPbBr_3$ film.

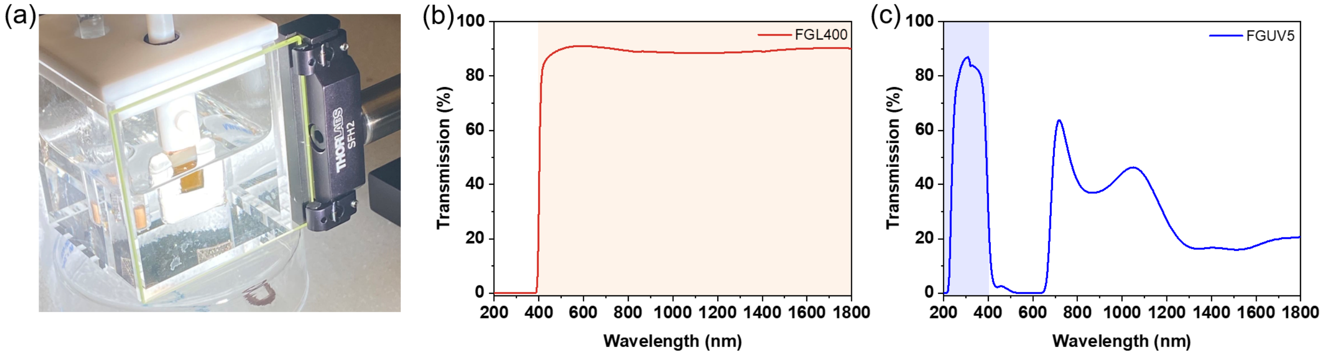

Supplementary Fig. 32 (a) Digital images of filter and test cell. Transmission UV-vis data of (b) long pass UV filter (>400 nm) and (c) band pass visible light filter (200-400 nm). Data was collected from *www.thorlabs.com.*

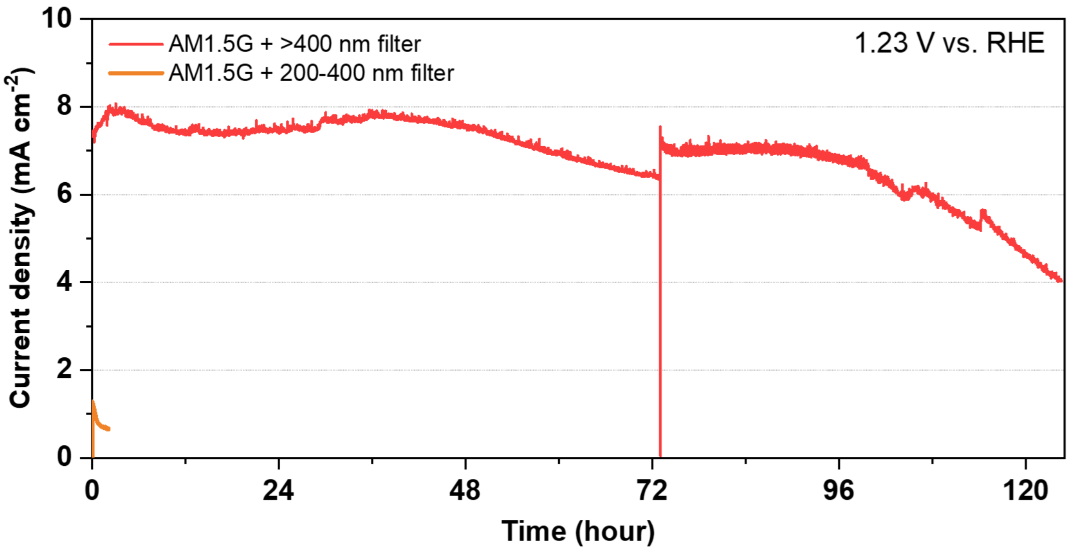

Supplementary Fig. 33 Chronoamperometric measurement of $FAPbBr_3$ photoanode at 1.23 V vs. RHE in 1.0 M KOH solutions (100 mW cm^{-2} AM 1.5G + UV/vis. filter).

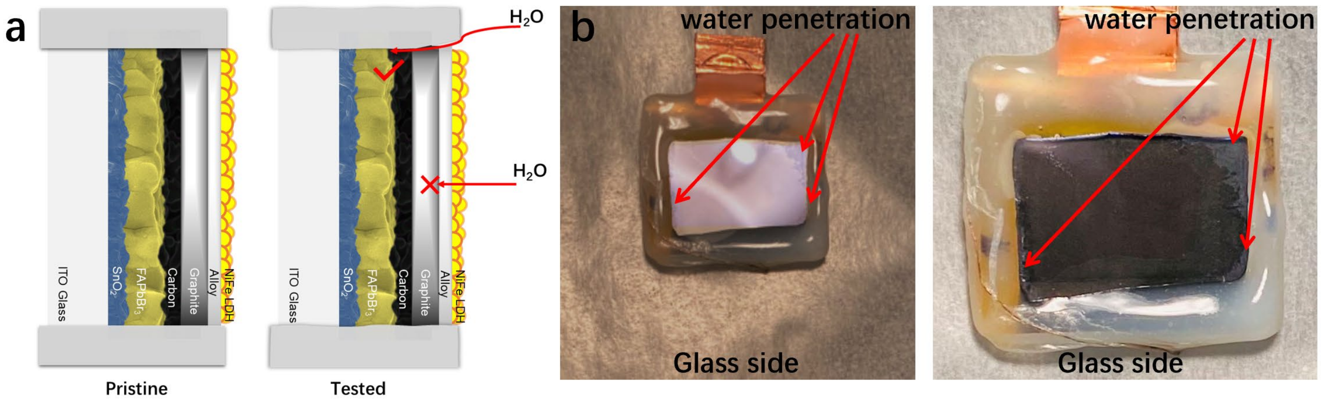

Supplementary Fig. 34 (a) Schematic illustration of device degradation due to seal failure. (b) Digital images of the photoanode after operation time of over 125 hours. The edge of the device clearly demonstrates the degradation of the perovskite layer due to water penetration.

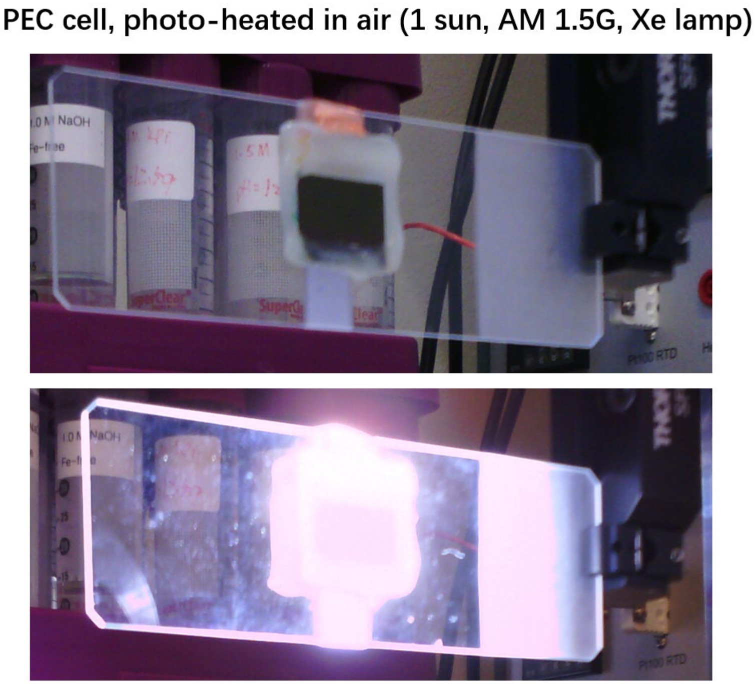

Supplementary Fig. 35 Digital images of photo-heated $FAPbBr_3$ photoanode in ambient air.

Supplementary Note 5: Temperature-dependent characterizations.

The thermodynamic driving force of a given reaction varies with electrolyte acidity when pH-independent reference electrodes such as Ag/AgCl and Hg/HgO are used. The slight difference of pH at different temperatures may lead to errors in the calculated overpotentials. To avoid potential errors when using the Hg/HgO reference electrode, the reference electrode was directly calibrated with RHE in as-prepared 1.0 M KOH with different temperatures. As shown in ***Supplementary Fig. 36***, the difference between Hg/HgO and RHE ($E_{RHE}^{Hg/HgO}$) are measured to be from 0.919 V to 0.900 V when the temperature changed from 1 to 50°C, respectively. The correlation between theoretical water oxidation potential (*E*) and temperature (*T*) can be expressed in the form of a linear equation in the temperature range from 10 to 50°C: $E = -0.0008436T + 1.48$ (***Supplementary Fig. 37***).[33] The overpotentials could be calculated using the following ***Supplementary Equation 5***:

$$\eta^{H_2O} = E_{Hg/HgO}^{read} + E_{RHE}^{Hg/HgO} - (-0.000843T + 1.48)\ \mathrm{V} \quad (5)$$

The difference of overpotentials in 1.0 M KOH/1°C and 1.0 M KOH/50°C is approximately 20 mV, which cannot be neglected; therefore, it is reasonable to compare the current densities at the same overpotential by using the abovementioned RHE correction. Then the relationship between apparent activation energy (*W*) and overpotential was obtained according to our previously report (***Supplementary Fig. 38***).[33]

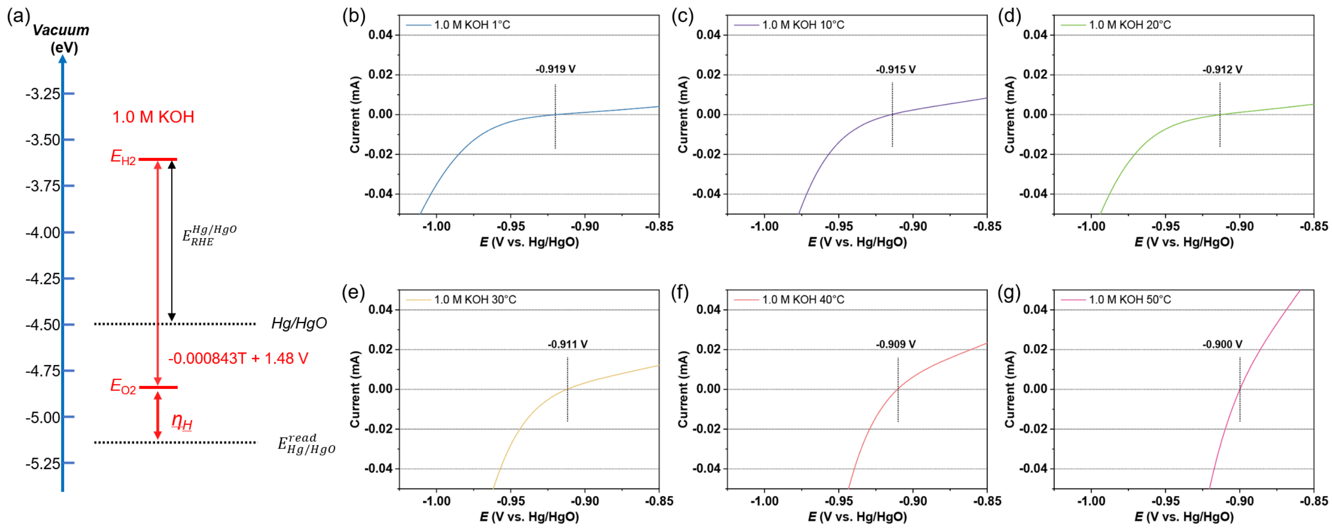

Supplementary Fig. 36 (a) Calculation of overpotential for water oxidation in 1.0 M KOH solutions. (b-g) Current–potential curves of Pt wire in H_2-saturated 1.0 M KOH solutions, used for calibration of the Hg/HgO electrode with respect to RHE at different temperatures; scan rate: 10 mV s^{-1}.

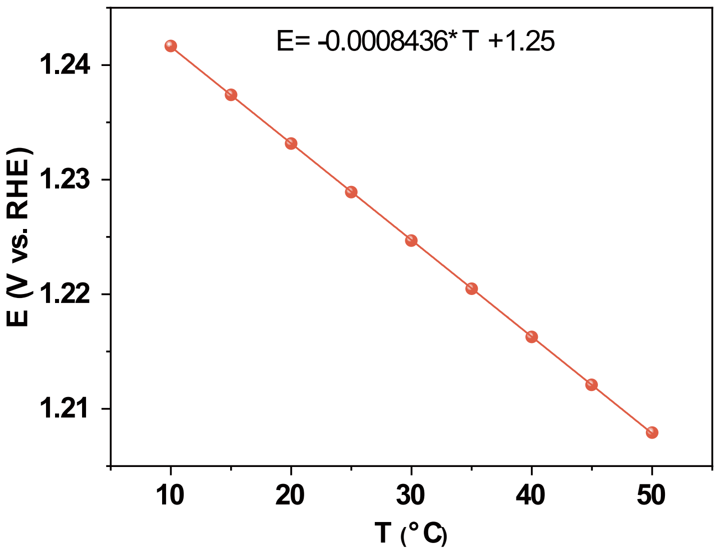

Supplementary Fig. 37 The correlation between theoretical water oxidation potential (E_{OER}) and temperature (T).

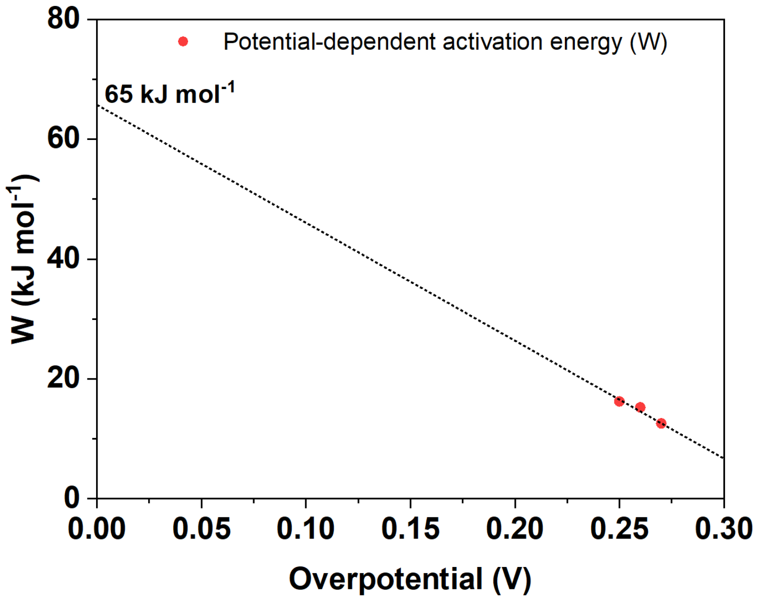

Supplementary Fig. 38 Plots of the overpotential-dependent activation energy (*W*) against the reaction overpotential.

The temperature effect of $FAPbBr_3$ solar cell is studied by a lab-made device. The cell was heated by circulating heated water through the heating jacket of a metal thermal pad. As shown in the infrared image in ***Supplementary Fig. 39***, the solar cell was uniformly heated by the metal thermal pad. The exposed area is controlled to be 0.8 cm^{-2}, and the light source is a Xe arc lamp with AM 1.5G filter.

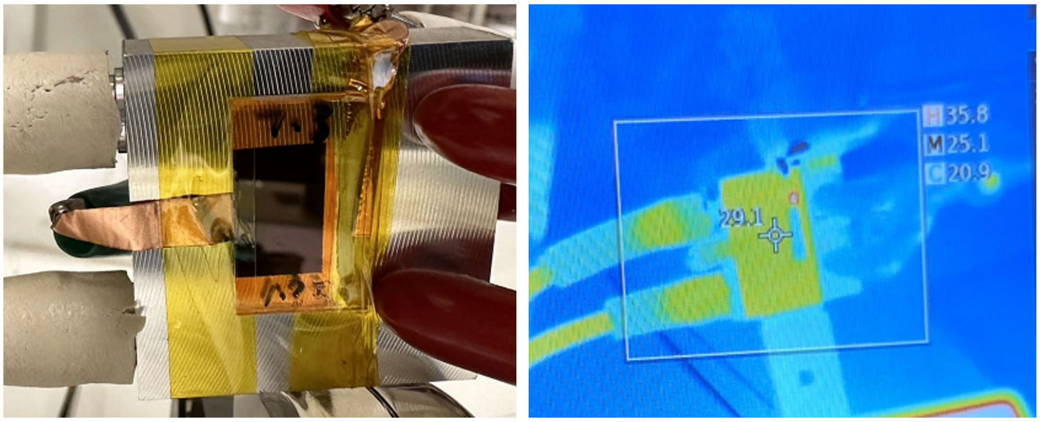

Supplementary Fig. 39 Lab-made temperature control setup for solar cell.

During the investigation of the temperature effect of $FAPbBr_3$ photoanode, the electrolyte is heated by circulating heated water through the heating jacket of a double-walled photoelectrochemical cell. The *J*-*V* curve is one of the key parameters used to describe photoelectrode materials. In many reports, the voltage is typically expressed in terms of the RHE value,[16, 34, 35, 36] while in the overpotential representation, voltage is expressed in terms of its potential value relative to the target reaction potential. By using overpotential to represent the *J*-*V* curve, the current response of a photovoltaic material at a specific potential can be more accurately depicted, allowing for a better understanding of the electrochemical properties and performance of the material under the same driving force.

In our case, when using RHE to represent the *J*-*V* curve, both the onset potential and catalytic current of the photoanode have been significantly improved under high-temperature conditions (***Supplementary Fig. 40***). However, this decrease in onset potential may be due to a reduction in the theoretical water oxidation potential (E_{OER}) for water oxidation and may not reflect the actual influence of temperature on the charge separation and catalytic processes (***Supplementary Fig. 37***). Therefore, expressing the *J*-*V* curve in terms of water oxidation potential $(E-E_{OER})_T$ at specific temperature (i.e., overpotential for electrochemical reaction) provides a more reasonable and intuitive way to describe the electrochemical characteristics and behavior of photovoltaic materials.

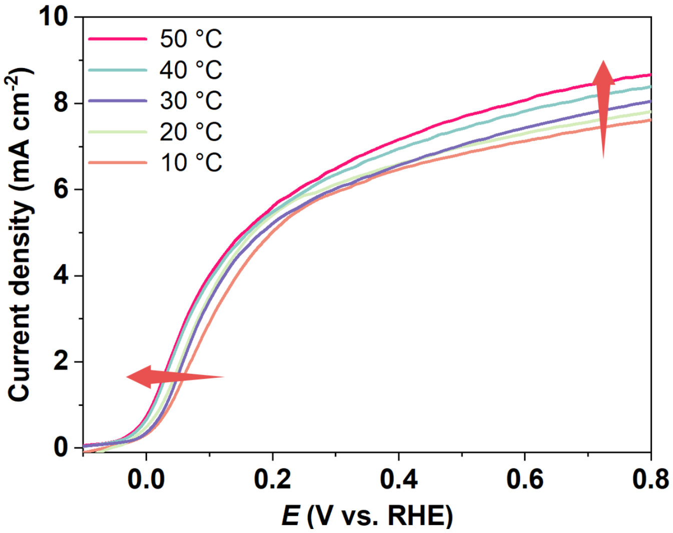

Supplementary Fig. 40 Temperature-dependent LSV curves of $FAPbBr_3$ photoanode under light illumination (AM 1.5G, 100 mW cm^{-2}), data is presented in RHE scale.

Supplementary Note 6: Summary of photothermal effects on different photocatalytic water splitting systems.

The water-splitting reaction that produces hydrogen from water is a complex process that involves the transfer of multiple protons and electrons. Moreover, the high thermodynamic requirements for O-O bond formation makes it challenging for single-junction semiconductor photoelectrodes used in most photoelectrochemical (PEC) cells to drive the water-splitting reaction directly without the assistance of bias.[37, 38, 39, 40] Despite using high band gap semiconductor materials (over 2.0 eV) such as $BiVO_4$ and Fe_2O_3, oxide semiconductor-based PEC face difficulty in providing the sufficient driving force for the total water splitting reaction due to the mismatching of the energy band structure. Therefore, identifying a single semiconductor material that can simultaneously generate sufficient photovoltage and effectively harvest a large portion of the solar spectrum remains an ongoing and complex challenge in the field of PEC. To achieve unassisted PEC cells, a typical schematic named photoanode-photocathode (or Z-scheme) tandem PEC cell is presented in ***Supplementary Fig. 41a***. Z-scheme PEC enables the absorption of a broader spectrum and provides sufficient photovoltage for water splitting by combining the individual photovoltages of the two photoelectrodes in the system. When the system is heated by incident light after specific modification,[16, 34] the simultaneously enhanced catalytic kinetics for each photoelectrodes lead to an increased operation current, which can indirectly expand the spectral utilization range (***Supplementary Fig. 41d***). For photovoltaic-electrochemical (PV-EC) devices in which the light absorber is separated from the electrolyte, the PV and EC components can be treated as two independent parts, which allows for greater flexibility in terms of modularization and optimization (***Supplementary Fig. 41b***). However, single-junction solar cells cannot provide enough photovoltage for electrocatalysts (over 1.6 V), thus typically multijunction structures is required to achieve bias-free PV-EC systems. Solar panels are inevitably heated under illumination, which represents 75-96% of the total absorbed solar energy,[41] resulting in a reduction in output performance and lifetime.[42, 43, 44, 45] As shown in ***Supplementary Fig. 41e***, the decrease in FF and V_{oc} directly results in a reduction of operational current; thus effective photovoltaic panel cooling strategy is highly important for the future solar hydrogen application.

The photovoltaic materials-based photoelectrochemical (PVM-PEC) configuration overcomes the disadvantage of panel heating in the PV-EC system (***Supplementary Fig. 41c***). Our work demonstrates that perovskite-based PEC can fully utilize the photothermal effect, like PEC, to maximize solar energy utilization. As presented in ***Supplementary Fig. 41f***, the photothermal effect of PVM-PEC can be analyzed like a solar cell, where the enhanced catalytic performance at high temperature compensates performance loss in FF and V_{OC}, which delivers an increased photocurrent under higher bias. Photothermal heat can be efficiently transferred to the catalyst layer through a reasonable structural design, avoiding performance and stability losses caused by heat accumulation while also accelerating the reaction kinetics. The integrated design also reduces the complexity of the system. The advanced PV materials used in PVM-PEC deliver significantly better performance than those used in traditional PEC, making PVM-PEC highly competitive with traditional PEC. Moreover, short charge migration distance in PVM-PEC configuration facilitates the scalability without the problem of linearly increasing series resistance as the PV area increases, which outperform the PV-EC system.

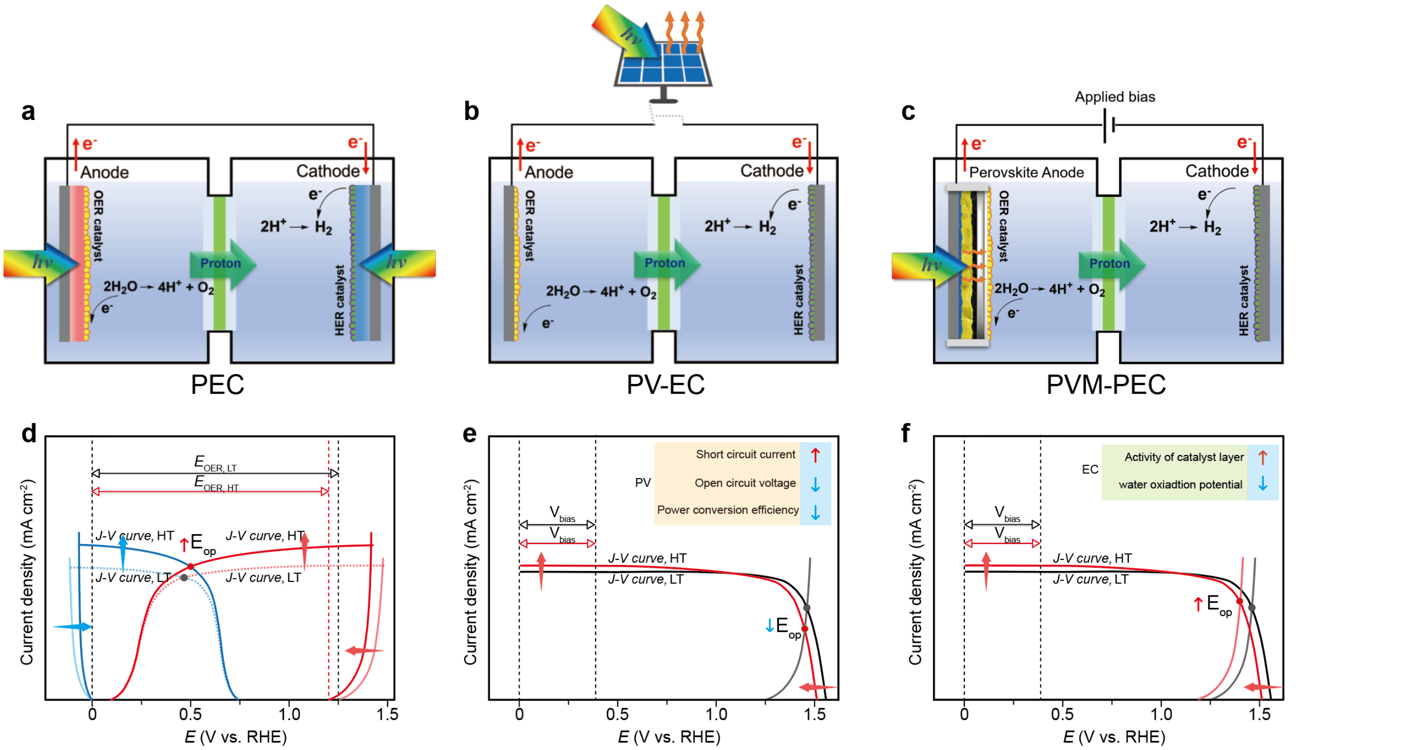

Supplementary Fig. 41 Schematic diagrams of (a) Z-scheme PEC, (b) PV-EC, and (c) PVM-PEC systems. Scheme diagrams of temperature effects on LSV curves and operation points (E_{op}) of (d) Z-scheme PEC, (e) PV-EC, and (f) PVM-PEC devices.

Supplementary References

1. Poli I, *et al.* Graphite-protected $CsPbBr_3$ perovskite photoanodes functionalised with water oxidation catalyst for oxygen evolution in water. *Nat Commun* **10**, 2097 (2019).

2. Kim TG, *et al.* Monolithic Lead Halide Perovskite Photoelectrochemical Cell with 9.16% Applied Bias Photon-to-Current Efficiency. *ACS Energy Lett* **7**, 320-327 (2022).

3. Wang M, *et al.* High-Performance and Stable Perovskite-Based Photoanode Encapsulated by Blanket-Cover Method. *ACS Appl Energy Mater* **4**, 7526-7534 (2021).

4. Liu Y, Kim BJ, Wu H, Boschloo G, Johansson EMJ. Efficient and Stable $FAPbBr_3$ Perovskite Solar Cells via Interface Modification by a Low-Dimensional Perovskite Layer. *ACS Appl Energy Mater* **4**, 9276-9282 (2021).

5. Liu Y, *et al.* Flexible Lead Bromide Perovskite Solar Cells. *ACS Appl Energy Mater* **3**, 9817-9823 (2020).

6. Zhang Y, Liang Y, Wang Y, Guo F, Sun L, Xu D. Planar $FAPbBr_3$ Solar Cells with Power Conversion Efficiency above 10%. *ACS Energy Lett* **3**, 1808-1814 (2018).

7. Liang J, *et al.* All-Inorganic Perovskite Solar Cells. *J Am Chem Soc* **138**, 15829-15832 (2016).

8. Yang H, *et al.* Improving the performance of water splitting electrodes by composite plating with nano-SiO_2. *Electrochimica Acta* **281**, 60-68 (2018).

9. Lu X, Zhao C. Electrodeposition of hierarchically structured three-dimensional nickel–iron electrodes for efficient oxygen evolution at high current densities. *Nat Commun* **6**, 6616 (2015).

10. Yu X, Yang P, Chen S, Zhang M, Shi G. NiFe Alloy Protected Silicon Photoanode for Efficient Water Splitting. *Adv Energy Mater* **7**, 1601805 (2017).

11. Guo B, *et al.* Facile Integration between Si and Catalyst for High-Performance Photoanodes by a Multifunctional Bridging Layer. *Nano Letters* **18**, 1516-1521 (2018).

12. Luo Z, *et al.* Multifunctional Nickel Film Protected n-Type Silicon Photoanode with High Photovoltage for Efficient and Stable Oxygen Evolution Reaction. *Small Methods* **3**, 1900212 (2019).

13. Liu Z, *et al.* Tailored NiFe Catalyst on Silicon Photoanode for Efficient Photoelectrochemical Water Oxidation. *J Phys Chem C* **124**, 2844-2850 (2020).

14. Cai Q, Hong W, Jian C, Liu W. A high-performance silicon photoanode enabled by oxygen vacancy modulation on NiOOH electrocatalyst for water oxidation. *Nanoscale* **12**, 7550-7556 (2020).

15. Li F, *et al.* Electroless Plating of NiFeP Alloy on the Surface of Silicon Photoanode for Efficient Photoelectrochemical Water Oxidation. *ACS Appl Mater Interfaces* **12**, 11479-11488 (2020).

16. He B, *et al.* General and Robust Photothermal-Heating-Enabled High-Efficiency Photoelectrochemical Water Splitting. *Adv Mater* **33**, 2004406 (2021).

17. Li F, *et al.* A Cobalt@Cucurbit[5]uril Complex as a Highly Efficient Supramolecular Catalyst for Electrochemical and Photoelectrochemical Water Splitting. *Angew Chem Int Ed* **60**, 1976-1985 (2021).

18. Wang S, *et al.* In Situ Formation of Oxygen Vacancies Achieving Near-Complete Charge Separation in Planar $BiVO_4$ Photoanodes. *Adv Mater* **32**, 2001385 (2020).

19. Zeng G, Deng Y, Yu X, Zhu Y, Fu X, Zhang Y. Ultrathin g-C3N4 as a hole extraction layer to boost sunlight-driven water oxidation of BiVO4-Based photoanode. *J Power Sources* **494**, 229701 (2021).

20. Zhang X*, et al.* Engineering Single-Atomic Ni-N4-O Sites on Semiconductor Photoanodes for High-Performance Photoelectrochemical Water Splitting. *J Am Chem Soc* **143**, 20657-20669 (2021).

21. Wang Y*, et al.* Highly Efficient Photoelectrochemical Water Splitting with an Immobilized Molecular Co4O4 Cubane Catalyst. *Angew Chem Int Ed* **56**, 6911-6915 (2017).

22. Chen H*, et al.* Integrating Low-Cost Earth-Abundant Co-Catalysts with Encapsulated Perovskite Solar Cells for Efficient and Stable Overall Solar Water Splitting. *Adv Funct Mater* **31**, 2008245 (2021).

23. Hoang MT, Pham ND, Han JH, Gardner JM, Oh I. Integrated Photoelectrolysis of Water Implemented On Organic Metal Halide Perovskite Photoelectrode. *ACS Appl Mater Interfaces* **8**, 11904-11909 (2016).

24. Nam S, Mai CTK, Oh I. Ultrastable Photoelectrodes for Solar Water Splitting Based on Organic Metal Halide Perovskite Fabricated by Lift-Off Process. *ACS Appl Mater Interfaces* **10**, 14659-14664 (2018).

25. Daboczi M, Cui J, Temerov F, Eslava S. Scalable all-inorganic halide perovskite photoanodes with> 100 h operational stability containing Earth-abundant materials. *ChemRxiv* **Cambridge: Cambridge Open Engage**, This content is a preprint and has not been peer-reviewed. (2023).

26. Tao R, Sun Z, Li F, Fang W, Xu L. Achieving Organic Metal Halide Perovskite into a Conventional Photoelectrode: Outstanding Stability in Aqueous Solution and High-Efficient Photoelectrochemical Water Splitting. *ACS Appl Energy Mater* **2**, 1969-1976 (2019).

27. Cho H-H*, et al.* A semiconducting polymer bulk heterojunction photoanode for solar water oxidation. *Nat Catal* **4**, 431-438 (2021).

28. Rhee R*, et al.* Unassisted overall water splitting with a solar‐to‐hydrogen efficiency of over 10% by coupled lead halide perovskite photoelectrodes. *Carbon Energy* **5**, e232 (2023).

29. Xue H, Brocks G, Tao S. First-principles calculations of defects in metal halide perovskites: A performance comparison of density functionals. *Physical Review Materials* **5**, 125408 (2021).

30. Jeon TH, Moon G-h, Park H, Choi W. Ultra-efficient and durable photoelectrochemical water oxidation using elaborately designed hematite nanorod arrays. *Nano Energy* **39**, 211-218 (2017).

31. Liu B*, et al.* A BiVO4 Photoanode with a VOx Layer Bearing Oxygen Vacancies Offers Improved Charge Transfer and Oxygen Evolution Kinetics in Photoelectrochemical Water Splitting. *Angew Chem Int Ed* **62**, e202217346 (2023).

32. Dong G, Hu H, Huang X, Zhang Y, Bi Y. Rapid activation of Co3O4 cocatalysts with oxygen vacancies on TiO2 photoanodes for efficient water splitting. *J Mater Chem A* **6**, 21003-21009 (2018).

33. Yang H*, et al.* Intramolecular hydroxyl nucleophilic attack pathway by a polymeric water oxidation catalyst with single cobalt sites. *Nat Catal* **5**, 414-429 (2022).

34. Zhou C, Zhang L, Tong X, Liu M. Temperature Effect on Photoelectrochemical Water Splitting: A Model Study Based on BiVO4 Photoanodes. *ACS Appl Mater Interfaces* **13**, 61227-61236 (2021).

35. Dias P, Lopes T, Andrade L, Mendes A. Temperature effect on water splitting using a Si-doped hematite photoanode. *J Power Sources* **272**, 567-580 (2014).

36. Jiang W*, et al.* Stress-induced BiVO4 photoanode for enhanced photoelectrochemical performance. *Appl Catal B* **304**, 121012 (2022).

37. Chi J*, et al.* Recent advancements in bismuth vanadate photoanodes for photoelectrochemical water splitting. *Materials Today Chemistry* **26**, 101060 (2022).

38. Kim JH, Hansora D, Sharma P, Jang J-W, Lee JS. Toward practical solar hydrogen production – an artificial photosynthetic leaf-to-farm challenge. *Chem Soc Rev* **48**, 1908-1971 (2019).

39. Chen D, Xie Z, Tong Y, Huang Y. Review on BiVO4-Based Photoanodes for Photoelectrochemical Water Oxidation: The Main Influencing Factors. *Energy & Fuels* **36**, 9932-9949 (2022).

40. Meng L, Li L. Recent research progress on operational stability of metal oxide/sulfide photoanodes in photoelectrochemical cells. *Nano Research Energy* **1**, e9120020 (2022).

41. Li R, Shi Y, Wu M, Hong S, Wang P. Photovoltaic panel cooling by atmospheric water sorption–evaporation cycle. *Nature Sustainability* **3**, 636-643 (2020).

42. Bredemeier D, Walter D, Herlufsen S, Schmidt J. Lifetime degradation and regeneration in multicrystalline silicon under illumination at elevated temperature. *AIP Advances* **6**, 035119 (2016).

43. Jordan DC, Kurtz SR. Photovoltaic Degradation Rates—an Analytical Review. *Progress in Photovoltaics: Research and Applications* **21**, 12-29 (2013).

44. Natarajan SK, Mallick TK, Katz M, Weingaertner S. Numerical investigations of solar cell temperature for photovoltaic concentrator system with and without passive cooling arrangements. *International Journal of Thermal Sciences* **50**, 2514-2521 (2011).

45. Skoplaki E, Palyvos JA. On the temperature dependence of photovoltaic module electrical performance: A review of efficiency/power correlations. *Solar Energy* **83**, 614-624 (2009).
